# Evolutionary dynamics in plastomes and mitogenomes of diatoms

**DOI:** 10.1371/journal.pone.0331749

**Published:** 2025-09-05

**Authors:** Aimee Caye G. Chang, Mailor W. W. Amaral, Megan Greenwood, Catherine Ikudaisi, Jingchun Li, Sarah E. Hamsher, Scott Miller, Patrick Kociolek

**Affiliations:** 1 Museum of Natural History and Department of Ecology and Evolutionary Biology, University of Colorado, Boulder, Colorado, United States of America; 2 Department of Biological Sciences, College of Science, University of Santo Tomas, Manila, Philippines; 3 Department of Biology and Annis Water Resources Institute, Grand Valley State University, Allendale and Muskegon, Michigan, United States of America; 4 Division of Biological Sciences, University of Montana, Missoula, Montana, United States of America; Franklin & Marshall College, UNITED STATES OF AMERICA

## Abstract

Diatoms are pivotal in global oxygen, carbon dioxide, and silica cycling, contributing significantly to photosynthesis and serving as fundamental components in aquatic ecosystems. Recent advancements in genomic sequencing have shed light on their evolutionary dynamics, revealing evolutionary complex genomes influenced by symbiotic relationships and horizontal gene transfer events. By analyzing publicly available sequences for 120 plastomes and 70 mitogenomes, this paper aims to elucidate the evolutionary dynamics of diatoms across diverse lineages. Gene losses and pseudogenes were more frequently observed in plastomes compared with mitogenomes. Overall, gene losses were particularly abundant in the plastomes of *Astrosyne radiata*, *Toxarium undulatum,* and *Proboscia* sp. Frequently lost and pseudogenized genes were *acpP*, *ilv*, *serC*, *tsf*, *tyr*C, *ycf42* and *bas1*. In mitogenomes, *mttB*, *secY* and *tatA* genes were lost repeatedly across several diatom taxa. Analysis of nucleotide substitution rates indicated that, in general, mitogenomes were evolving at a more rapid rate compared to plastomes. This is contrary to what was observed in synteny analyses, where plastomes exhibited more structural rearrangements than mitogenomes, with the exception of the genus *Coscinodiscus* and one group of species within *Thalassiosira*.

## Introduction

Diatoms play crucial roles in the global cycling of oxygen, carbon dioxide, and silica [1,2]. Comprising 20–40% of global oxygen production and carbon fixation through photosynthesis, their efficiency in this process surpasses many other photoautotrophs [3,4]. Found at the base of the food chain in most aquatic ecosystems, they serve as fundamental components within these ecological systems [5]. As an important interface between the atmosphere, terrestrial environment and aquatic ecosystem, diatoms have been used extensively to document impacts on aquatic ecosystems due to change in the atmosphere and global climate change [6–8], as well as human induced perturbations originating in terrestrial environments [9].

Understanding diatom biology and phylogeny has been based upon various aspects of their cell cycle [10, 11], along with the morphology of soft [cytoplasmic, e.g., 12–14] and hard parts [their glass cell walls, e.g., 15–17]. More recently, data from single genes [18], multiple genes from a single organelle [19,20] or multiple organelles [21,22], and entire genomes [23–25] have been used to study a myriad of aspects of diatom biology and systematics. The first whole organellar genomes were sequenced from diatoms beginning in 1995 (from the chloroplast genome of the diatom *Odontella sinensis* Z67753). Since that time, over 120 taxa of diatoms have had their chloroplast genomes sequenced and over 70 taxa have had their mitochondria sequenced. Based on genome features on publicly available databases, diatom plastomes range from 111,539 bp to 216,580 bp, while mitogenomes range from 32,777 bp to 177,614 bp. GC content analysis revealed ranges of 28.40 to 35.80% and 21.60 to 35.00% for plastomes and mitogenomes, respectively. Moreover, gene content showed plastomes have counts ranging from 194 to 238 total genes, while mitogenomes have 52–65 genes. Phylogenies derived from the plastome and mitogenome of diatoms showed concordance between the two organelles [20]. When compared with various diatom classification schemes from the past 150 years, based on cytoplasmic (i.e., the number, position and division of chloroplasts, the position and division of the nucleus in the cell cycle, number of gametes produced, position of oil droplets in the cytoplasm), frustule, and molecular data, it was evident that a natural classification (i.e., one comprised of monophyletic groups; see [26] for the group has been lacking.

Beyond phylogeny, organellar genomes offer the opportunity to explore a myriad of other aspects of diatom biology. Because organellar genomes have a lower recombination rate, they are more convenient to use in tracing evolutionary relationships [27]. Throughout evolutionary history, nuclear, plastid or mitochondrial genomes undergo DNA alterations, ranging from small nucleotide substitutions to significant rearrangements [28]. Comparative gene studies have highlighted localized changes like nucleotide substitutions, gene insertions and deletions [29]. However, recombination stands out as a mechanism capable of inducing substantial transformations, including gene loss, duplication, rearrangement, and horizontal gene transfer [30]. Exploring the frequencies and mechanisms of these genomic changes would give valuable insights in understanding the biological evolution of eukaryotes.

Diatom nuclear genomes are described as chimeric due to their history of secondary endosymbiosis [31]. The extant set of genes shared by all diatom genomes was acquired from one of the following sources: an ancient heterotrophic host, a green and a red algal endosymbiont, and various bacteria through horizontal gene transfer events. Since green and red algae are subdivisions of the Archaeplastida, originating from primary endosymbiotic events, diatom genomes also incorporate genes from cyanobacteria and their hosts [32]. Diatoms also have extensive genic and genomic rearrangements occurring in chloroplast and mitochondrial genomes [33,34].

The availability of extensive data for both plastomes and mitogenomes together offers the opportunity to assess organellar evolution through comparison of their evolutionary rates of these organelles overall for both diatoms as a whole and for individual clades to assess organellar evolution. Based on the data from the two organelles and other approaches, here we can begin to explore the origin and evolution of the group and the changes that led to their remarkable success in nearly every aquatic environment on earth.

## Materials and methods

### Taxon selection

The whole chloroplast and mitochondrial genomes of diatoms were sourced from the NCBI GenBank, selecting taxa based on the availability of complete genomes online as of September 2023. Genomes that had not been published in journals by the time of genome acquisition and the start of the analysis, or those lacking annotations where only nucleotide sequence data (fasta files) were provided, were excluded from the analyses. For the selection of taxa used in the analysis, the date of collection and actual sources of the strains were not considered. Strains that were part of a culture collection or long-term culture preservation were still included in the analysis. This study includes a total of 120 plastomes and 70 mitogenomes of diatoms, with five taxa *Triparma laevis*, *Aureococcus anophagefferens*, *Heterosigma akashiwo*, *Sargassum plagiophyllum*, and *Nannochloropsis oculata* serving as outgroups. All GenBank accession numbers are listed in **S1 Table**.

### Gene extraction, alignment and phylogenetic trees

A set of 78 chloroplast and 25 mitochondrial protein-coding genes, common to 120 plastomes and 70 mitogenomes, along with five outgroups, were identified and extracted using Geneious Prime v2023.2.1 (https://www.geneious.com) [35]. Each gene underwent individual alignment using the MAFFT software v1.5.0 plug-in in Geneious Prime using default settings. The alignment was refined using trimAl v1.5.0 [36] to ensure nucleotide bases corresponding to amino acids were aligned and removing large gaps with ambiguous regions that had low nucleotide similarities within the alignments. The aligned and trimmed genes were then concatenated, resulting in a final alignment length of 61,931 bp for chloroplasts and 16,826 bp for mitochondria (https://doi.org/10.5061/dryad.76hdr7t5j).

The alignments based on the shared protein-coding genes were uploaded to the CIPRES Science Gateway [37], where Bayesian Inference (BI) trees were generated using the Mr. Bayes phylogeny software. BI phylogenetic reconstruction was performed using four MCMC chains of one million generations with sampling frequency of every 1,000 generations, and the first 25% generations were discarded as burn-in. The chloroplast and mitochondrial Bayesian trees containing posterior probabilities are provided as supplementary files (S1 File and S2, respectively).

### Gene Content Analysis

The analysis of gene presence, absence, and pseudogenization employed Dollo parsimony in MacClade v4.08 [38], utilizing the encoding scheme of 1, 0, and 2 to denote present, absent, and pseudogenized (i.e., those located but with stop codons throughout their sequence) states, respectively. After completing the character matrix, each character history was mapped and traced to the pre-generated phylogenetic tree based on aligned protein-coding gene sequences. The Dollo parsimony model was used to assume that gene losses from the genomes are more probable than gene gains, and to suggest that functional genes are more likely to transition into pseudogenes rather than the opposite. A supplementary file presenting the gene presence, absence and pseudogenes across all diatom taxa for plastomes and mitogenomes was provided (S2 Table).

### Revalidation of gene losses

**Fig 1:**
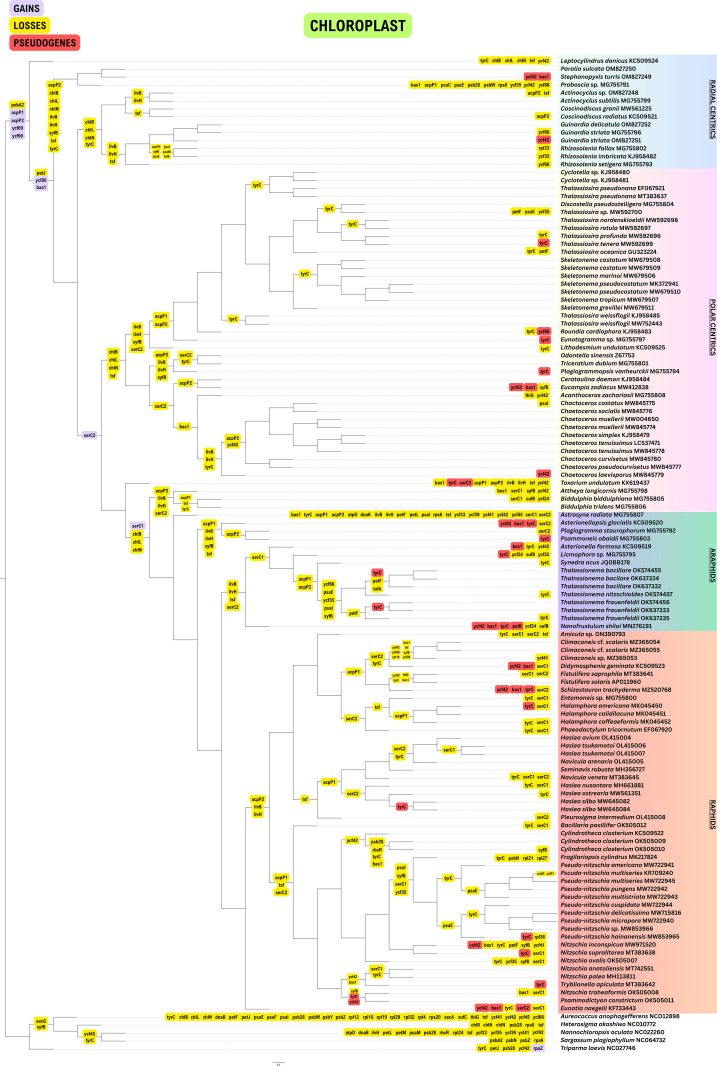
Gene loss and gain events in plastomes of 120 diatom taxa using Dollo parsimony. The four major groups of diatoms are highlighted.

**Fig 2:**
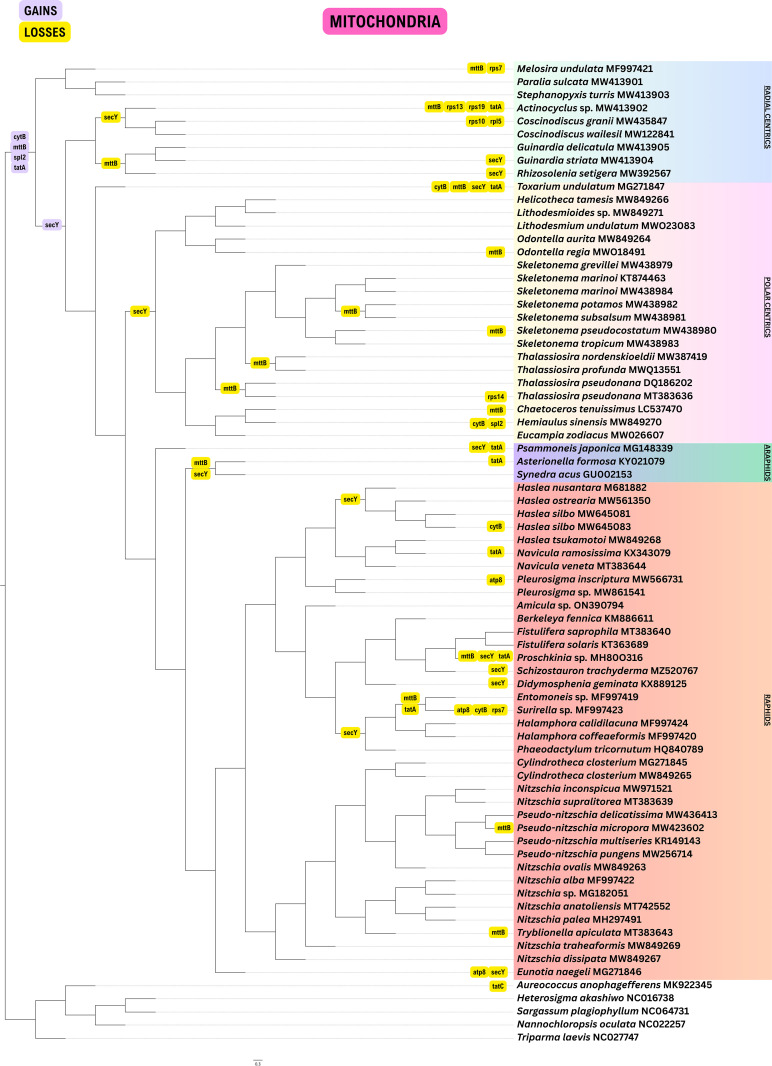
Gene loss and gain events in mitogenomes of 70 diatom taxa using Dollo parsimony. The four major groups of diatoms are highlighted.

Genomes deposited in NCBI GenBank may contain annotation errors or missing gene data, which can lead to misinterpretations regarding gene losses and gains. In fact, this was the case for every one of the published organellar genomes we analyzed. To address this, the authors included a confirmatory step to verify whether genes were actually absent or simply missing due to annotation issues. Thus, all genomes were re-annotated using multiple reference genomes with complete gene sets to ensure annotation completeness. All plastomes and mitogenomes were re-annotated using the GeSeq tool on the CHLOROBOX web server (https://chlorobox.mpimp-golm.mpg.de/geseq.html) [39]. For both organelles, protein-coding genes, rRNAs, and tRNAs were annotated based on BLAT searches against reference genomes, selecting one representative genome from each major monophyletic diatom group (Figs 1-2) to ensure coverage of close evolutionary relatives. This approach allows annotation to capture genes conserved across lineages while minimizing bias from overly distant taxa. Annotations were supplemented by HMMER profile searches against the organelle reference databases provided in GeSeq. Additionally, tRNAs were predicted using tRNAscan-SE v2.0.7. Default parameters were applied for all steps, and the resulting annotations were used directly in subsequent gene content and synteny analyses. The re-annotated files are available at https://doi.org/10.5061/dryad.70rxwdc7b.

### Evolutionary rates estimation

Pairwise distances based on nucleotide substitutions were estimated using MEGA X v10.2.6 [40]. The mean distances across all sequences were derived from sequence alignments of shared 78 protein-coding genes (PCGs) for plastomes and 25 PCGs for mitogenomes. The analysis includes genes common to all diatom taxa used in this study. In accordance with distance measure selection guidelines, the Jukes-Cantor model with nucleotide substitutions (*d*) value within 0.05 < d < 0.3 was chosen for both the chloroplast and mitochondrion.

### Synteny analysis

Genomic rearrangements of diatom genera with more than one representative for both plastomes and mitogenomes were analyzed using progressiveMauve v1.1.3 in Geneious Prime software [41] by estimating locally collinear blocks (LCBs). Structural variations in plastomes were visualized after removing one copy of the inverted repeat (IR_B_) and aligning the large single copy (LSC), small single copy (SSC) and IR_A_ regions in linearized sequences of all genomes.

### Statistical analysis

Statistical analyses were conducted in the R programming environment [42], considering a significance level α = 0.05 (*p* < 0.05). Potential statistical differences in evolutionary rates between chloroplast and mitochondrial genomes based on pairwise distances of nucleotide substitutions were compared using two-factor variance analysis (two-way ANOVA), and the post-hoc Tukey’s test when significant. Type III mixed model analysis was used, accounting for the unbalanced design of the data, such as the different number of plastomes and mitogenomes.

## Results

### Gene loss events in plastomes

Seventy-eight (78) conserved genes were present in all diatom plastomes and the rest of the genes that were lost and pseudogenized were mapped in Fig 1. Most of the genes coding for ATP synthase (*atpA, atpB, atpD, atpE, atpF, atpG, atpH, atpI*) were present in all 120 plastomes of diatoms except for *atpD* which has been lost in *Astrosyne radiata*. The peroxiredoxin gene *bas1* was lost 34 times and pseudogenized in eight diatom genera – *Asterionellopsis*, *Asterionella*, *Didymosphenia*, *Eunotia, Nanofrustulum, Schizostauron* and *Stephanopyxis*. Of the four light-independent chlorophyll-*a* biosynthesis genes (*chlB, chlI, chlL, chlN*), only *chlI* was present in all diatoms. The genes *chlB, chlL* and *chlN* were absent in most diatom lineages and retained only in *Toxarium undulatum*. DNA replication gene *dnaK* was absent in *Astrosyne*.

Photosystem I genes *psaA, psaB, psaD, psaF, psaJ* and *psaL* were present in all diatoms but *psaC* is absent in *Proboscia, psaM* was not found in *Rhizosolenia imbricata* and *R. fallax*, and *psaE* and *psaI* were lost multiple times in different lineages. Most photosystem II (PSII) genes (*psbA*, *psbB, psbC, psbD, psbE, psbF, psbH, psbI, psbJ, psbK, psbL, psbT, psbV, psbX, psbY, psbZ*) were found in all diatoms, but other PSII genes were lost in some taxa. Both *Fragilariopsis cylindrus* and *Pseudo-nitzschia multiseries* (KR709240) had lost *psbN* gene and *psbW* was not present in *Proboscia*.

Genes coding for cytochrome *b/f* complexes (including *petA, petB, petD*, *petG, petL*, *petM* and *petN*) were present in most diatoms, with *petB* pseudogenized in *Nanofrustulum shiloi* and *petL* lost only in *Astrosyne*. *petJ* was lost in all diatoms except for *Leptocylindrus* and the *petF* gene was lost multiple times across the diatom tree of life.

In diatoms, both the RuBisCO large subunit (*rbcL*) gene and its small subunit gene (*rbcS*) are located in the chloroplast, unlike in plants where *rbcS* is usually encoded in the nucleus [43]. The *rbcL* and *rbcS* genes are present in all diatoms. However, its transcriptional regulator gene *rbcR* has been lost in two strains of *Cylindrotheca*. Also present in all diatoms were RNA polymerase genes *rpoA, rpoB*, *rpoC1* and *rpoC2*. One RNA polymerase gene, *rpoZ*, was absent in all diatoms and found only in one of the outgroup taxa, *Triparma laevis*. The acetolactate synthase large and small subunits, *ilvB* and *ilvH* genes, were also lost multiple times in different lineages.

Twenty-one large subunit ribosomal ribonucleic acid (LSU rRNA) genes were present in all diatoms: *rpl1, rpl2, rpl3, rpl4, rpl5, rpl6, rpl11, rpl12, rpl13, rpl14, rpl16, rpl18, rpl20, rpl22, rpl24, rpl23, rpl29, rpl31, rpl32, rpl34, rpl35*. Other LSU rRNA genes were lost sporadically in various lineages, but notably, *rpl19* was absent in all *Climaconeis*, *Fragilariopsis* lost two genes (*rpl2* and *rpl27*), *R. fallax* lost *rpl33* and *Pseudo-nitzschia hainanensis* lost *rpl36*. Sixteen small subunit ribosomal ribonucleic acid (SSU rRNA) were present in all diatom taxa: *rps2, rps3, rps4, rps5, rps7, rps9, rps10, rps11, rps12, rps13, rps14, rps16, rps17, rps18, rps19* and *rps20*. The gene *rps6* was not found in *Astrosyne* and *rps8* was lost in *Proboscia*.

### Gene loss events in mitogenomes

Compared to genomic events occurring in plastomes, pseudogenization of genes was uncommon and gene losses were rare in mitogenomes (Fig 2).

ATP synthase genes, *atp6* and *atp9* were present in all 70 mitogenomes of diatoms, but *atp8* gene was lost independently 3 times in *Eunotia*, *Pleurosigma* and *Surirella*. All complex IV cytochrome oxidase genes (*cox1, cox2, cox3*) are present in all lineages, as well as all complex I NADH dehydrogenase genes (*nad1, nad2, nad3, nad4, nad4L, nad5, nad6, nad7, nad9, nad11*). For complex III ubichinolcytochrome-c reductase genes, *cob* was present in all diatoms, but the *cytB* gene was lost independently 4 times in *Hemialus*, *Haslea silbo* (MW645083), *Surirella* and *Toxarium*.

Four ribosomal large subunit genes (*rpl2*, *rpl6*, *rpl14*, *rpl16*) were present in all diatoms. The *rpl5* gene was lost in *Coscinodiscus granii*. Out of the 10 *rps* genes, only five were present in all mitogenomes – *rps3, rps4*, *rps8, rps11* and *rps12*. *Melosira* and *Surirella* lost *rps7*, *Actinocyclus* lost both *rps13* and *rps19*, *rps10* was absent in *C. granii* and *rps14* was not found in *Thalassiosira pseudonana* (MT383636).

The mitochondrial inner membrane translocase protein gene, *tatA*, and the other protein translocase subunit *secY* gene were retained sporadically across diatom lineages. The gene encoding the TatC protein, a key component of the twin-arginine translocation (Tat) system, is conserved across all diatom lineages, unlike the genes for other translocase proteins – *tatA* and *secY*. The trimethylamine methyltransferase gene, *mttB*, was lost independently 15 times.

### Evolutionary rates based on nucleotide substitutions

**Fig 3:**
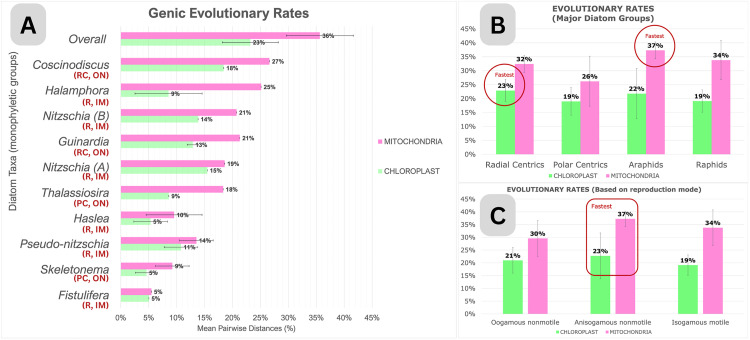
Evolutionary rate estimates of a) chloroplast and mitochondrial genomes from various genera based on the overall mean of pairwise distances of nucleotide substitutions (R2 = 0.571); b) based on major diatom groups (R2 = 0.551); c) based on reproduction mode using Jukes-Cantor model (R2 = 0.511). Detailed ANOVA statistical values are represented in Table 1. Genera are categorized based on monophyletic groups. [RC = radial centrics, PC = polar centrics, A = araphids, R = raphids, ON=oogamous nonmotile, AN = anisogamous nonmotile, IM = isogamous motile].

The Analysis of Variance (two-way) for diatom evolutionary rates based on nucleotide substitutions showed statistically significant differences among the various factors analyzed (Table 1), with mitogenomes evolving faster than plastomes (Fig 3). The association *Chloroplast + Mitochondria* examines the organellar evolutionary rates separately, comparing their individual dynamics. The analysis of *Chloroplast + Mitochondria* within specific groupings (*Genus* or *Diatom Groups* or *Reproduction mode*) investigates whether their evolutionary rates differ significantly based on the unique characteristics of each grouping. The association between *Chloroplast + Mitochondria + Grouping* assesses overall trends in organelle evolution, determining if there are significant differences in their rates when considering the entire dataset.

**Table 1:** Analysis of Variance (two-way) for diatom evolutionary rates based on nucleotide substitutions. Statistically significant values (*p* < 0.05) are in bold.

	DF	F	*p*-value
**Genus**			
Chloroplast + Mitochondria	1	3.205	0.099
Genus	12	21.905	**<0.0001**
Chloroplast + Mitochondria * Genus	12	6.300	**<0.0001**
**Diatom Groups**			
Chloroplast + Mitochondria	1	2.526	0.210
Phylogenetic Group	3	1.124	0.463
Chloroplast + Mitochondria * Group	3	72.311	**<0.0001**
**Reproduction Mode**			
Chloroplast + Mitochondria	1	2.374	0.263
Reproduction	3	0.204	0.831
Chloroplast + Mitochondria * Reproduction	3	111.468	**<0.0001**

Across all genera represented by more than one species that have sequence data for both organelles (Fig 3a), there is no significant difference in genic evolutionary rates between chloroplasts and mitochondria when analyzed collectively (*p* = 0.099). However, significant differences in evolutionary rates exist between genera (**p* *< 0.0001) and between chloroplast and mitochondria when analyzed within each specific genus (*p* < 0.0001), with mitochondria evolving faster (Table 1).

Among the 10 diatom groups, *Coscinodiscus* exhibited the fastest mitochondrial genome evolution, followed by *Halamphora*. Evolving at similar rates were *Nitzschia* group B (*N. anatoliensis*, *N. palea*) and *Guinardia*, followed by *Nitzschia* group A (*N. inconspicua*, *N. supralitorea*), *Thalassiosira*, *Pseudo-nitzschia*, *Haslea*, and *Skeletonema*. The slowest rate of mitogenome evolution was observed in *Fistulifera*.

In contrast, the chloroplast genome evolutionary rates showed a different pattern across genera. *Coscinodiscus* still evolved the fastest, but was followed by *Nitzschia* group A, then *Nitzschia* group B and *Guinardia*. *Pseudo-nitzschia* came next, with *Halamphora* and *Thalassiosira* evolving at similar rates. As with the mitogenomes, the slowest plastome evolution was observed in *Haslea*, *Skeletonema*, and *Fistulifera*.

Rates across four major diatom groups (sensu [44], radial centrics, polar centrics, araphids and raphids – which includes non-monophyletic groups) were also analyzed (Fig 3b). The overall comparison of chloroplast and mitochondria across all groups did not show a significant difference (*p* = 0.210). Additionally, there were no significant differences in evolutionary rates between the groups themselves (**p* *=* *0.463). However, significant differences in evolutionary rates were observed between chloroplast and mitochondria when analyzed within each group (**p* *< 0.0001), except for polar centrics. Comparisons within plastomes revealed radial centrics as the most rapidly evolving group, characterized by the highest nucleotide substitution rates. In contrast, mitogenomic evolution presented a distinct pattern, with araphids emerging as the fastest-evolving group.

Categorizing diatoms according to their reproductive modes unveiled a more uniform trend (Fig 3c), with no significant differences in evolutionary rates among the reproductive groupings (*p* = 0.831). Additionally, the overall comparison of chloroplast and mitochondria rates did not show a significant difference (*p* = 0.263). However, significant differences in evolutionary rates were observed between chloroplasts and mitochondria when analyzed within each specific reproductive mode (*p* < 0.0001). Across both plastomes and mitogenomes, anisogamous nonmotile diatoms emerged as the group undergoing the most rapid evolution.

### Synteny analysis of Locally Collinear Blocks (LCBs)

**Fig 4:**
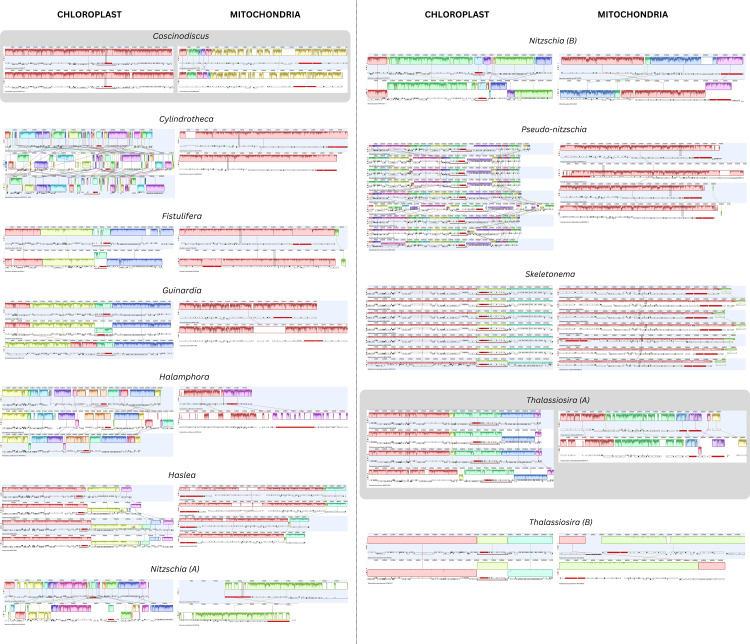
Gene order comparison of plastomes and mitogenomes represented with more than one taxon. Subgroups within genera represent monophyletic groups. Highlighted in gray boxes are the exceptions where more rearrangements were observed in the mitogenomes than plastomes. Alignment was performed after removing one copy of the inverted repeat, followed by the generation of locally collinear blocks (LCBs) using progressiveMAUVE.

Gene order comparisons of 12 monophyletic diatom groups revealed that plastomes generally have more genomic rearrangements (e.g., inversions and translocations) than mitogenomes resulting in inversions and translocations of gene blocks (Fig 4). The only exceptions were *Coscinodiscus* (radial centric) and a group of *Thalassiosira* (polar centric), wherein the mitogenomes were more structurally rearranged, with both having 7 LCBs in their mitogenomes and fewer LCBs (1 and 5 LCBs, respectively) in the plastomes. However, it is important to note that the number of LCBs does not directly correspond to the actual number of genomic rearrangement events, as a single event – such as an inversion – can result in the detection of multiple LCBs. For instance, in *Coscinodiscus*, an inversion of three adjacent LCBs combined with one translocated block resulted in the identification of seven separate LCBs. While attempts were made to infer specific rearrangement events from the LCB patterns, this was not feasible in certain cases, such as *Cylindrotheca* and *Pseudo-nitzschia*, where the high number of LCBs and extensive fragmentation made it difficult to confidently reconstruct the underlying rearrangement history.

*Cylindrotheca* has the most rearranged plastomes with 40 LCBs but only one LCB was observed across its mitogenomes. *Thalassiosira* group A, consisting of 4 taxa (*T. tenera, T. profunda, T. nordenskioeldii, T. rotula*), has 30 and 7 LCBs, respectively. The plastomes of *Pseudo-nitzschia* and *Nitzschia* group A (*N. inconspicua, N. supralitorea*) exhibited high structural rearrangements as well, featuring 18–20 LCBs, unlike their mitogenomes, which only showed a single LCB. *Nitzschia* group B (*N. anatoliensis*, *N. palea*) plastomes also showed several rearrangements with 10 LCBs in their plastomes and 4 LCBs in the mitogenomes. *Halamphora* plastomes have 13 LCBs, but their mitogenomes were more conserved in terms of gene order with only 2 LCBs observed. Compared to other diatom groups, the plastomes of *Haslea*, *Fistulifera*, *Guinardia*, *Skeletonema* and *Thalassiosira* group B (*T. pseudonana* DQ186202, MT383636) were less rearranged with only 3–5 LCBs, consistent with their mitogenomes that only showed ~ 1–3 LCBs. *Thalassiosira* and *Nitzschia* strains were categorized into two groups due to the current understanding that they are non-monophyletic. This separation of strains ensured that only monophyletic lineages were analyzed together.

## Discussion

### Gene loss events in diatom plastomes and mitogenomes

The comparative analysis of gene presence and loss across 120 diatom plastomes revealed intriguing patterns with some gene families more conserved than other gene groups. The retention of most ATP synthase genes across diverse diatom groups emphasizes their importance in cellular energy metabolism and their essential roles in ATP production via oxidative phosphorylation [45]. This finding wasn’t unexpected, considering previous reports indicating that even plastid genomes lacking photosynthetic capabilities typically conserve their ATP synthase complex genes [46]. However, the notable absence of the *atpD* gene in *Astrosyne* raises questions about the environmental pressures and evolutionary processes shaping gene retention and loss within this lineage. Some diatoms are also non-obligate photosynthetic species and therefore plastid reduction may naturally occur [47].

The *bas1* gene was lost in numerous diatom species, and this occurrence has also been observed in both red algal and the SAR (Stramenopiles, Alveolates, and Rhizaria) group plastid genomes [48,49]. Ruck *et al*. [50] also found pseudogene fragments in *Asterionellopsis*, *Asterionella*, *Eunotia* and *Didymosphenia*, suggesting more ongoing losses in various lineages. Further organellar genomic analysis is needed to determine if the antioxidative protection provided by *bas1* to plastomes has been lost, replaced, or transferred to the nucleus in some diatoms.

We also looked at the four genes involved in chlorophyll synthesis in diatoms. Only one gene, *chlI*, was found in all diatoms we studied. The other three genes (*chlB, chlL*, and *chlN*) were missing in all diatoms, except for one species, *Toxarium undulatum*, which retained these genes. It has been documented that the chlorophyll genes *chlB* (light-independent protochlorophyllide reductase), *chlL* and *chlN* (ATP-dependent Clp protease) serve as distinctive signature genes within the chloroplast genome across cyanobacteria, algae, bryophytes, pteridophytes, and gymnosperms [51] but surprisingly, has been lost in most diatoms. Diatoms likely lost the *chlB*, *chlL*, and *chlN* genes, which encode the subunits of dark-operative protochlorophyllide reductase (DPOR), due to their adaptation to oxygenic photosynthesis and reliance on the light-dependent NADPH:protochlorophyllide oxidoreductase (LPOR). DPOR, which catalyzes the reduction of protochlorophyllide in the absence of light, is highly sensitive to oxygen, and its function is compromised in oxygen-rich environments. In contrast, LPOR is oxygen-insensitive and performs the same reaction in the presence of light. As diatoms evolved under conditions with increasing atmospheric oxygen, they would have favored the oxygen-tolerant LPOR, rendering the oxygen-sensitive DPOR (and the associated *chlB*, *chlL*, and *chlN* genes) redundant, leading to their eventual loss [52].

The variability in the presence and loss of Photosystem I (PSI) and Photosystem II (PSII) genes across diatom lineages sheds light on the evolutionary dynamics of these key photosynthetic complexes. While core PSI genes are universally retained among diatoms, the sporadic losses of some *psa* and *psb* genes in multiple lineages suggests a complex history of gene retention and loss, further highlighting the evolutionary plasticity of photosystem genes in diatoms. The loss of *psa* and *psb* genes has been noted in higher photosynthetic organisms and has been identified as being transferred to the nuclear genome [51]. Therefore, additional diatom nuclear genomes will need to be sequenced to investigate and validate whether these gene transfer events also occur in diatoms.

The presence and absence of specific genes encoding cytochrome b/f complexes among diatoms also display intriguing evolutionary patterns. While *pet* genes remain conserved across all diatom species, the occurrence of pseudogenization and loss in some taxa emphasizes the complex nature of genomic evolution. The pseudogenization of *petB* in *Nanofrustulum* highlights a possible species-specific adaptation. Moreover, the recurrent loss of genes from the plastomes like *petL* and *petF* may suggest a complex interplay between genetic drift and selective pressures. These proteins are integral to the photosynthetic electron transport chain, and their deletion likely disrupts electron flow, probably altering some photosynthetic mechanisms. In addition, the retention of *petJ* solely in *Leptocylindrus* further accentuates the unique genomic landscapes influenced by evolution within diatom populations. According to Ruck *et al*. [50], *petJ* was transferred to the nucleus early in diatom evolution, after the split between *Leptocylindrus* and the rest of the diatoms. Functional *petF* and *petJ* genes may have undergone duplication or nuclear transfer [50,53,54]. Our study confirms the dual residency of the *petF* gene in both the plastome and nuclear genomes of *Thalassiosira pseudonana*, as proposed by Roy *et al*. [55].

Other key genetic elements within diatoms crucial for photosynthesis and protein synthesis display a diverse pattern of conservation and loss across different lineages. While the RuBisCO large and small subunit-associated genes remain universally present, the absence of their transcriptional regulator gene, *rbcR*, in some diatom taxa is interesting but requires more investigation. Similarly, the RNA polymerase widespread gene loss of *rpoZ* occurs across all diatoms; it is only found in the outgroup taxon *Triparma laevis*. The variability extends to ribosomal RNA genes, with sporadic losses observed in LSU rRNA genes across various lineages, notably within *Climaconeis* and *Fragilariopsis*. SSU rRNA genes, though largely conserved, also exhibit occasional losses. For highly variable and random losses, further analysis is required to discern whether they represent independent gains or instances of widespread losses across various lineages [56].

Overall, gene losses were prevalent in specific taxa such as *Astrosyne radiata*, *Toxarium undulatum*, and *Proboscia* sp., suggesting differential evolutionary and genomic diversification in these lineages. Notably, genes like *ilvB, ilvH*, and *syfB* have undergone multiple losses in these distantly related taxa. The repeated loss of *ilv* genes in diatoms, as also documented by Ruck *et al*. [50], Sabir *et al*. [57], and Yu *et al*. [27], suggests that these organisms may have found alternative functional solutions or that these genes are potentially unnecessary. According to Gile *et al*. [58], in diatoms lacking *syfB*, the synthesis of phe-tRNA is maintained through the dual targeting of the monomeric mitochondrial phenylalanyl-tRNA synthetase. The frequent loss of genes involved in essential cellular processes like elongation factor *tsf* and phosphoserine aminotransferase *serC1* and *serC2* shows the individual dynamic nature of diatom genomes. Particularly striking is the widespread loss of the cyclohexadienyl dehydrogenase *tyrC* gene and the acyl carrier protein genes *acpP1* and *acpP2* that needs to be explored further. Interestingly, independent pseudogenization events in *Nanofrustulum*, *Toxarium* and *Eunotia* highlight possible species-specific genomic evolution events. Moreover, the recurrent loss of various hypothetical chloroplast reading frame *ycf* genes shows the complex interaction between nuclear and chloroplast genomes in diatoms. According to Ševčíková *et al*. [59], some genes may appear absent in organisms, but they could actually be present in highly altered forms, making them difficult to identify using standard methods, highlighting the complexity of genetic evolution and the challenges in determining gene presence or absence based solely on sequence similarity.

Unlike the relatively rare occurrence of pseudogenization and gene losses in mitogenomes, plastomes seem to undergo more frequent alterations, suggesting a higher level of genome stability for the mitochondrion. Examining specific gene losses and retentions in mitogenomes sheds light on the selective pressures, or lack thereof, acting on mitochondrial genes within diatom lineages. For instance, the consistent presence of ATP synthase genes *atp6* and *atp9* across all 70 mitogenomes emphasizes their indispensable role in cellular energetics. However, the sporadic loss of the *atp8* gene, encoding a subunit of the F_1_F_0_-ATP synthase complex to aid in aerobic respiration and generate ATP within cells, hints at possible functional redundancy or lineage-specific adaptations. The loss of *atp8* in *Pleurosigma*, *Surirella* and *Eunotia* may not be surprising as it has also been reported to be transferred to the nucleus in some green algal lineages [60].

Similarly, the widespread occurrence of key respiratory complex genes such as *cox1, cox2*, and *cox3* underscores their fundamental importance in aerobic respiration. Yet, the selective loss of *cytB* across diatom lineages suggests unique evolutionary pathways shaped by ecological and physiological demands, or these changes could also be neutral or deleterious. Although gene duplication events have been reported in some diatoms – for example, two copies of the *cox1* gene in *Nitzschia traheaformis* and three copies in *Haslea tsukamotoi* [19] *–* our study focused only on the presence or absence of single-copy genes. However, we recommend that future studies explore gene duplication events more closely, as they are also important for understanding organellar genome evolution. The pattern of ribosomal RNA-associated gene losses highlights a degree of stochasticity in genomic evolution, with *rpl* and *rps* genes experiencing random losses across taxa. However, the consistent presence of certain SSU ribosomal protein genes like *rps3, rps4*, *rps8, rps11* and *rps12* hints at their essential functions in protein synthesis and ribosome assembly.

The observed losses of mitochondrial translocase protein genes, *tatA* and *secY*, suggest alternatives in mitochondrial protein import mechanisms. While the retention of *tatC* suggests its functional importance across diatom lineages, the losses of *tatA* hints at the possibility of losing its role in maintaining mitochondrial membrane integrity and protein translocation. In a study by Petrů *et al*. [61], they highlighted several instances where a *tat* gene (*tatC*) was lost from eukaryotic mitochondrial genomes. They further noted that unlike other genes, mitochondrial *tatC* was never transferred to the cell nucleus because of some physical characteristics such as hydrophobicity of the signal peptide, which is the primary obstacle to any nuclear transfer events. In diatom mitogenomes, genes such as *tatC*, *secY*, and *mttB* highlight the challenges associated with sparse gene annotations across public datasets. These genes are sometimes annotated separately and other times grouped together or given different names [62–64]. These differences likely come from the limited genomic resources available for diatoms, where annotation tools rely on distant or poorly defined reference genes. As in other resource-sparse systems, naming conventions are not yet standardized, and different research groups may use different terms for the same gene. This creates challenges when comparing gene content across species. To ensure accurate comparisons, we emphasize the importance of consistent gene naming and better reference data in future work.

It is important to note that some of the strains used in this study might have been in long term cultivation which could potentially affect the genome. It has been observed in *F. cylindrus* that long term artificial culture conditions have affected the gene expression of *rbcL* and *psbA* genes. Although it has been shown to affect gene expression, the effect of long-term cultivation in gene content and number remains limited and requires more research [65].

We also generally observed that tRNA and rRNA genes are highly conserved across diatom organellar genomes, showing limited variation among taxa. Therefore, our analyses focused primarily on protein-coding genes, which provided more informative signals for assessing evolutionary patterns and differences among species. However, we recognize the biological importance of tRNA and rRNA genes, and recommend that future studies explore their evolutionary dynamics in greater detail.

Uncertainties remain regarding the mechanisms behind gene losses and the strategies diatoms employ to adapt or compensate for the absence of certain genes. Thus, the gene losses and pseudogenization events discussed in this section require further investigation to uncover the reason behind all the gene loss events. An additional perspective that may help explain patterns of gene retention and loss in diatom organellar genomes is the control by epistasy of synthesis (CES) mechanism, which links gene expression to the assembly state of multi-protein complexes [66,67]. In this model, the translation of key subunits – often those initiating or regulating assembly – is post-transcriptionally regulated based on the availability of partner subunits. Thus, genes may be preferentially retained in organelles when their expression must be tightly coordinated with complex assembly. Conversely, genes that do not play a regulatory role in assembly or are not involved in post-transcriptional control may be more prone to loss, especially if their function can be compensated for by nuclear-encoded factors or if transcriptional regulation is no longer essential. Therefore, differential gene retention in diatom organelles may reflect not only functional roles but also regulatory importance, or lack thereof, in the context of complex assembly governed by CES.

### Comparison of evolutionary rates and synteny in plastomes and mitogenomes

Overall, genic differences are greater in the mitochondria of diatoms compared with chloroplasts. This trend is seen in some of the genera where there is more than a single species represented, such as *Coscinodiscus*, *Halamphora*, *Guinardia* and *Thalassiosira*, where the differences are statistically significant, and in genera such as *Haslea*, *Pseudo-nitzschia* and *Skeletonema* where the same trend is evident but not statistically different. Only in *Fistulifera* were the rates of evolution in the mitochondrion and chloroplast equal. Genic evolution was never more rapid in the chloroplast than in the mitochondrion in this study. A same trend occurs in the red algal genus *Porphyra* [68] and more distantly related lineages containing secondary red algae plastids, such as alveolates, coccolithophores and other stramenopiles, in which mitochondrial substitution rates are much higher than the chloroplast [69]. Substitution rates between the chloroplast and mitochondria organelles are variable in green algae, with similar numbers reported in *Chlamydomonas* and *Mesostigma* [70,71] and a higher substitution rate in the mitochondria of *Dunaliella* [71]. This is the opposite in higher plants, wherein nucleotide substitution rates are consistently greater in the chloroplast compared with mitochondria [72,73]. Overall, the rates of evolution in both the chloroplast and mitochondria of diatoms were much higher than recorded in green plants and bryophytes. The rate of base pair substitution was on par with the rates reported for primates and mammals [74]. Additionally, the number of plastids per cell in diatom lineages also varies, potentially influencing evolutionary rates, but this requires further investigation.

Our evolutionary rate analyses across the four major diatom groups – radial centrics, polar centrics, araphids, and raphids [44] – reflect broad morphological classifications rather than strict phylogenetic units. While radial centrics show the highest substitution rates in plastomes, araphids exhibit the fastest evolutionary rates in mitogenomes (Fig 3b). This pattern may be influenced by the limited number of available mitogenomes in this group. Consequently, the apparent rate of acceleration in araphid mitogenomes should be interpreted cautiously, and broader taxon sampling will be essential to clarify these dynamics.

While the rate of base-pair substitution was higher in the mitogenomes versus the plastomes of diatoms, the opposite was true in genomic structure. In diatoms, chloroplast structure had a high degree of structural differentiation as compared to mitochondria. Hamsher *et al*. [33] reported a greater structural variation in the chloroplasts among three species of *Halamphora* than what was found in bryophytes through angiosperms. In the results presented here, monophyletic groups within the genera *Thalassiosira* and *Nitzschia* (which are both paraphyletic genera [75,76]) show higher levels of structural variation in their chloroplasts among the species represented in them than was documented previously in *Halamphora*. The phenomenon of chloroplasts being structurally variable as compared to the mitogenomes in diatoms is also the opposite of the situation described in green plants. This reinforces the idea that chloroplast rearrangements are more frequent in algal groups compared to land plants [77,78]. And in red algae, structural differences between the chloroplasts and mitochondria mirror those in green plants, with the mitogenomes’ structure being more conserved than the plastomes but undergoing higher genic changes [79].

To provide additional context for interpreting evolutionary divergence, we compared genic and genomic identities across diatom taxa for both mitochondrial and chloroplast genomes (S1 Fig). While these values were generated using different approaches and are not directly comparable, they offer complementary perspectives: genic identity reflects divergence in coding regions, while genomic identity captures overall similarities or differences, including non-coding and intergenic regions. In some taxa, such as *Coscinodiscus* and *Guinardia*, relatively lower genomic identities suggest higher sequence divergence even when gene content or synteny appears conserved. This is particularly relevant in cases where synteny comparisons yield few or single LCBs, as high structural similarity may mask underlying sequence-level divergence. Thus, presenting and analyzing both genic and genomic identities helps clarify the degree of divergence among taxa and provides critical context for interpreting genome evolution across organelles – as presented in this study.

### Comparison of organellar evolution across diatoms with different features

Nakov *et al*. [80] compared species numbers, speciation rates and extinction rates among diatoms with different processes for sexual reproduction and ability for movement. Based on their searches of nomenclature databases [81,82], they concluded that the raphid diatoms (with isogamous reproduction and active motility) was the most species rich and had the fastest speciation and slowest extinction rates compared to other groups of diatoms (i.e., diatoms with oogamous reproduction and inability for movement and diatoms that are isogamous with little or no ability for motility). Some have suggested that features such as types of sexual reproduction or other physiological or ecological differences might be the cause for their success [80], reflected in higher rates of evolution. In the case of diatoms, there is no evidence from the organellar genomes to support their conclusion. For both plastomes and mitogenomes, raphid diatoms never had the highest substitution rates compared to other groups with different types of sexual reproduction or abilities for movement.

## Supporting information

S1 Table GenBank accession numbers. List of all NCBI GenBank accession numbers used in this study.
(XLSX)

S2 Table Gene presence and absence. A reference table listing genes showing gene absence (0-grey), presence (1-yellow), and pseudogenes (2-red). Genes shown in red font were conserved across all diatom taxa and were used for phylogenetic analysis and generating alignments.
(XLSX)

S1 Fig Genic vs. Genomic. Sequence divergence across diatom taxa for mitochondrial (top) and chloroplast (bottom) genomes, shown separately for genic and genomic regions. Genic identity reflects divergence in coding sequences, while genomic identity includes both coding and non-coding regions.
(PNG)

S1 File Plastome phylogenetic tree. Bayesian tree of 125 diatom plastomes with posterior probabilities.
(PDF)

S2 File Mitogenome phylogenetic tree. Bayesian tree of 75 diatom mitogenomes with posterior probabilities.
(PDF)

S3 File Synteny figures. Compilation of synteny comparisons (higher resolution images) for each genus.
(PDF)
