# Supplementary figures and images for: Evolutionary dynamics in plastomes and mitogenomes of diatoms
Source: PLoS One. 2025 Sep 5;20(9):e0331749. doi: 10.1371/journal.pone.0331749 (PMC12412971; doi:10.1371/journal.pone.0331749)

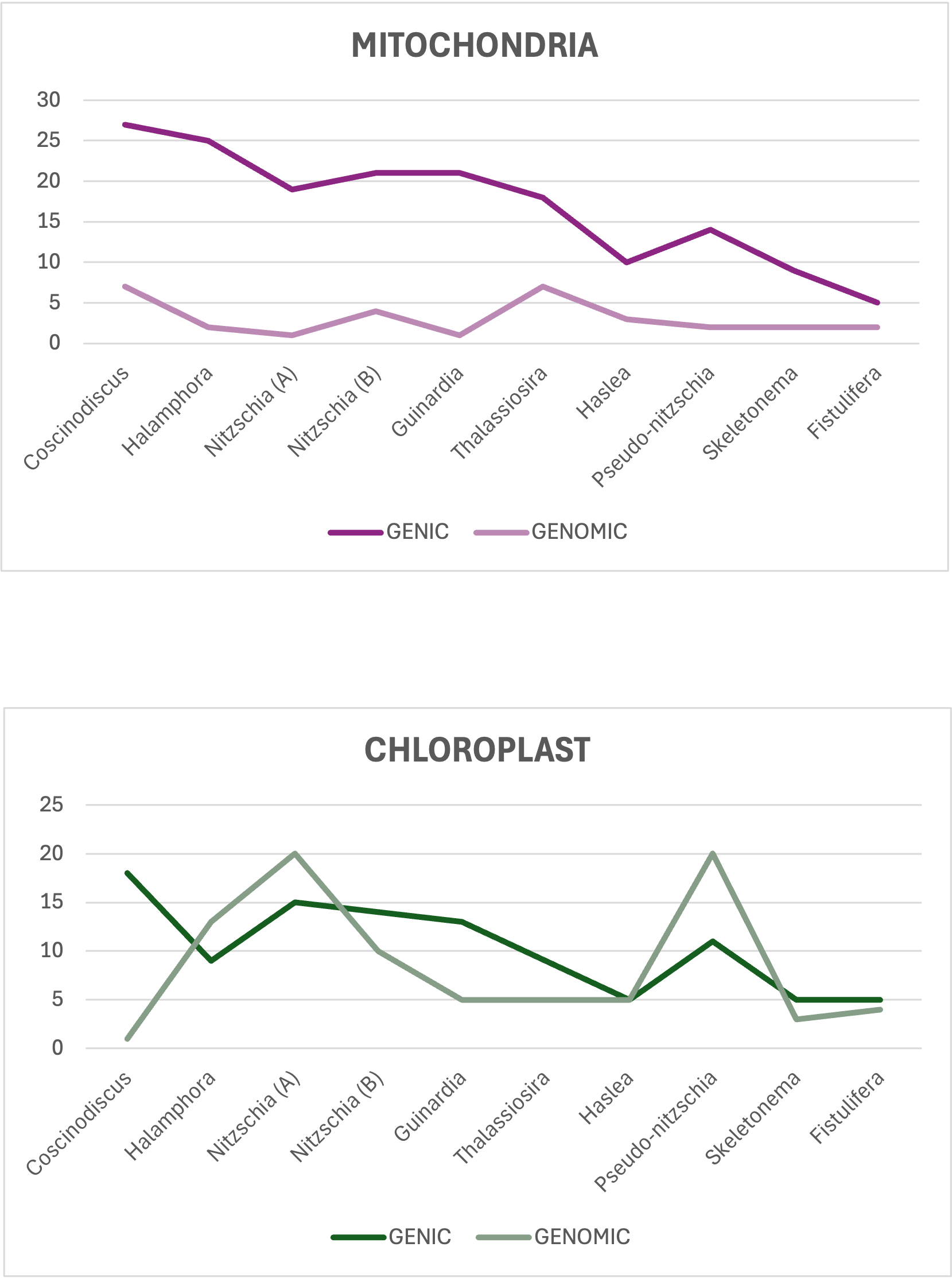

Supplement: S1 Fig — Sequence divergence across diatom taxa for mitochondrial (top) and chloroplast (bottom) genomes, shown separately for genic and genomic regions. Genic identity reflects divergence in coding sequences, while genomic identity includes both coding and non-coding regions. (PNG) [file pone.0331749.s003.png]

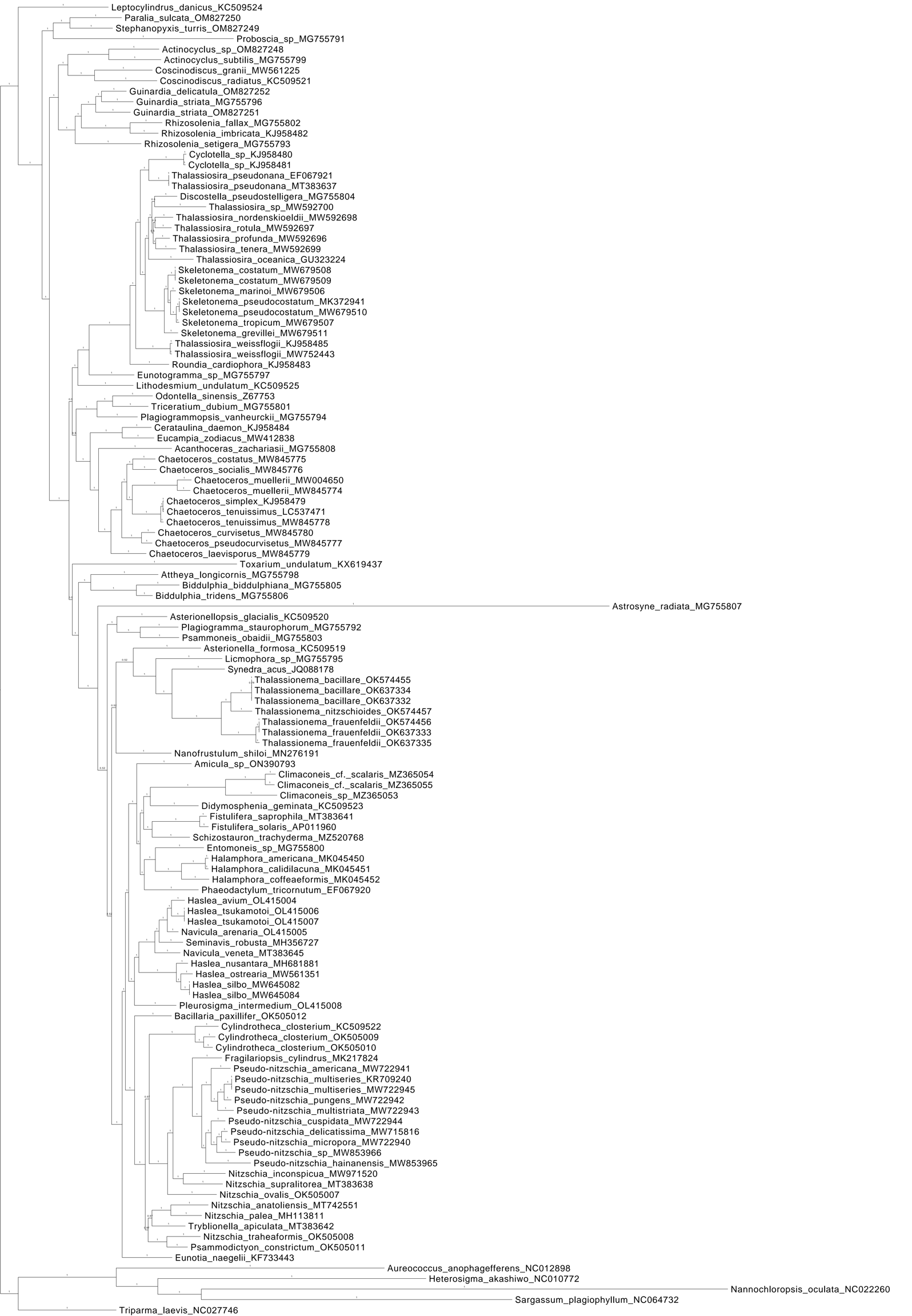

Supplement: S1 File — Bayesian tree of 125 diatom plastomes with posterior probabilities. (PDF) [file pone.0331749.s004.pdf]

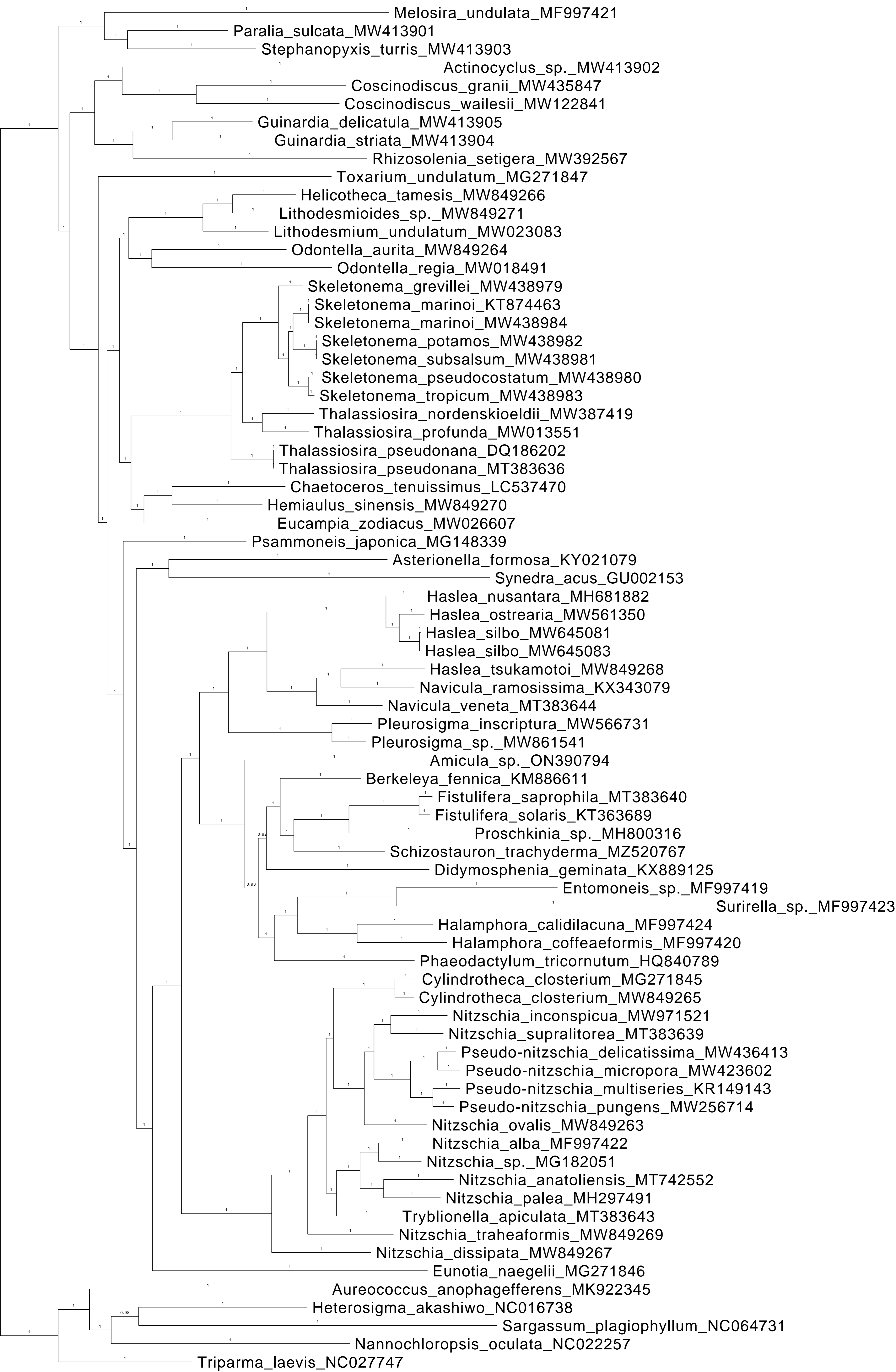

Supplement: S2 File — Bayesian tree of 75 diatom mitogenomes with posterior probabilities. (PDF) [file pone.0331749.s005.pdf]

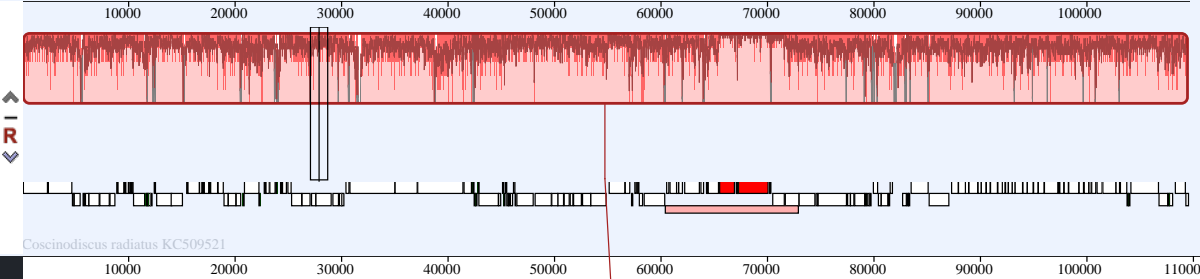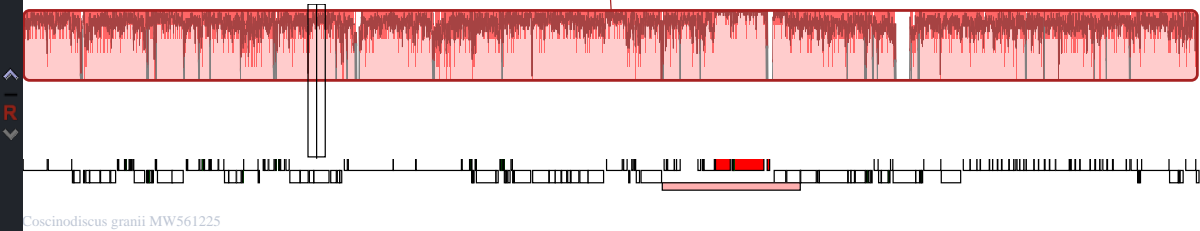

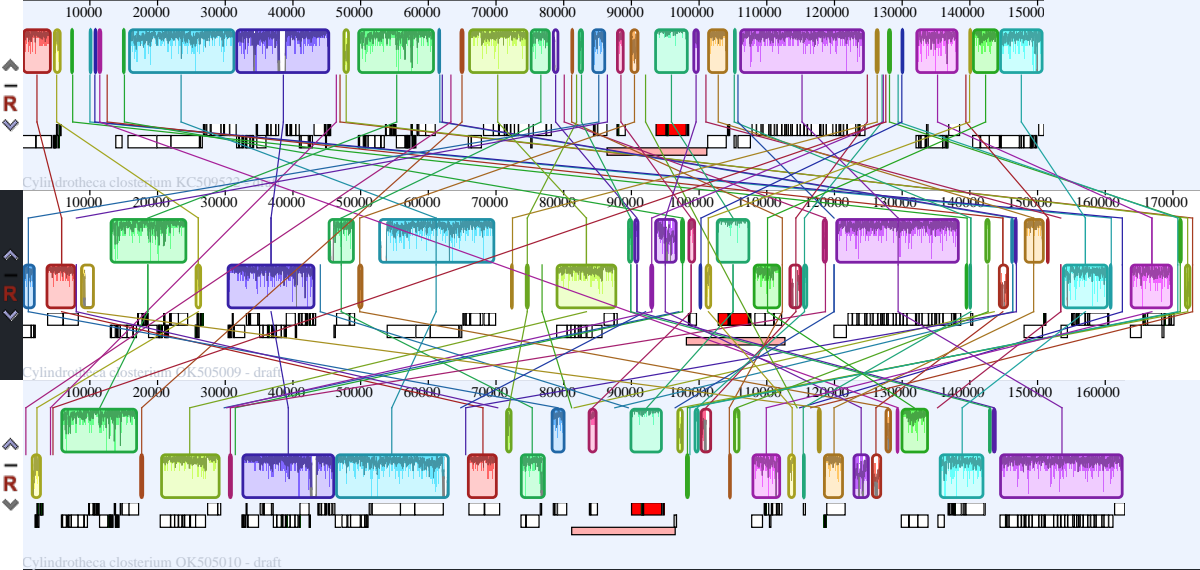

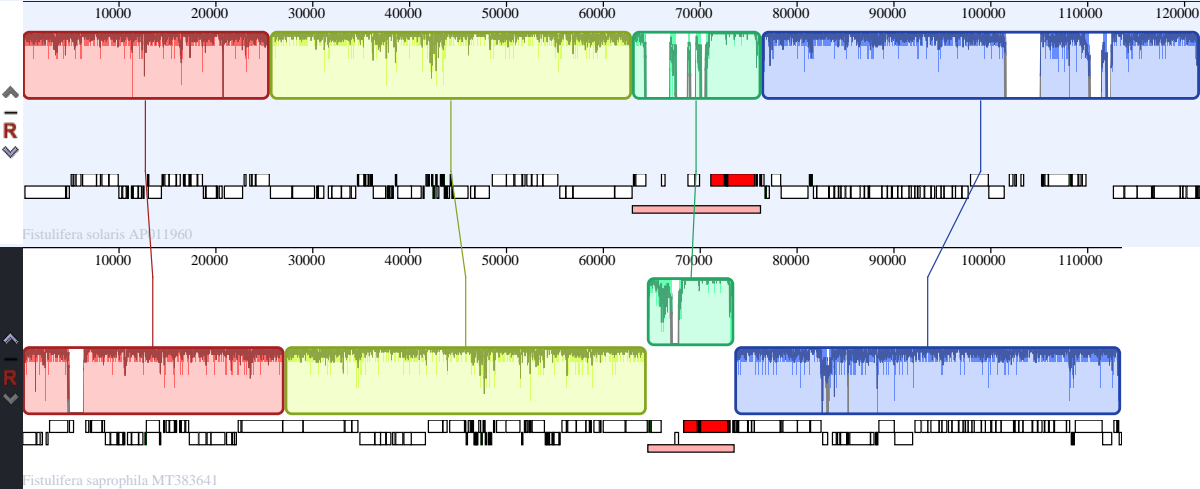

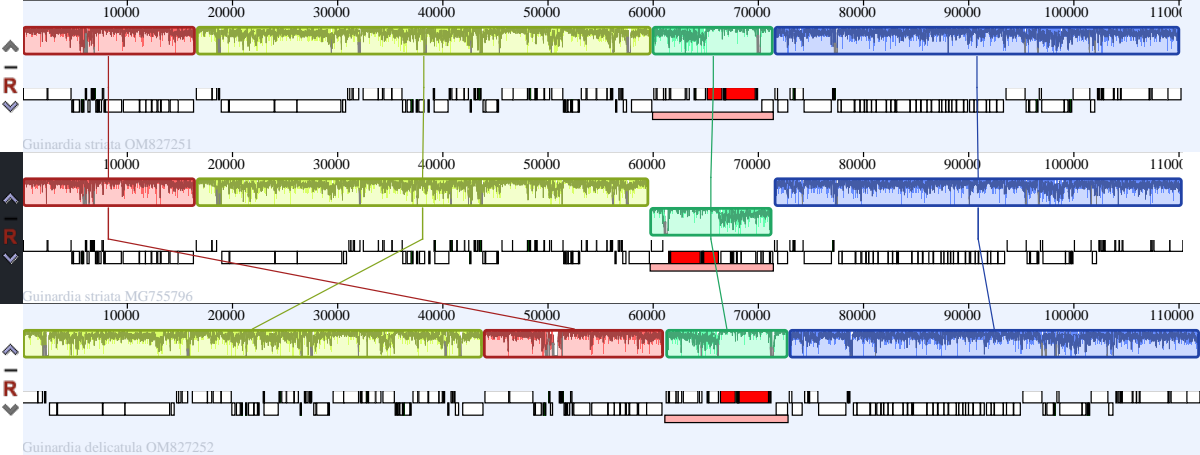

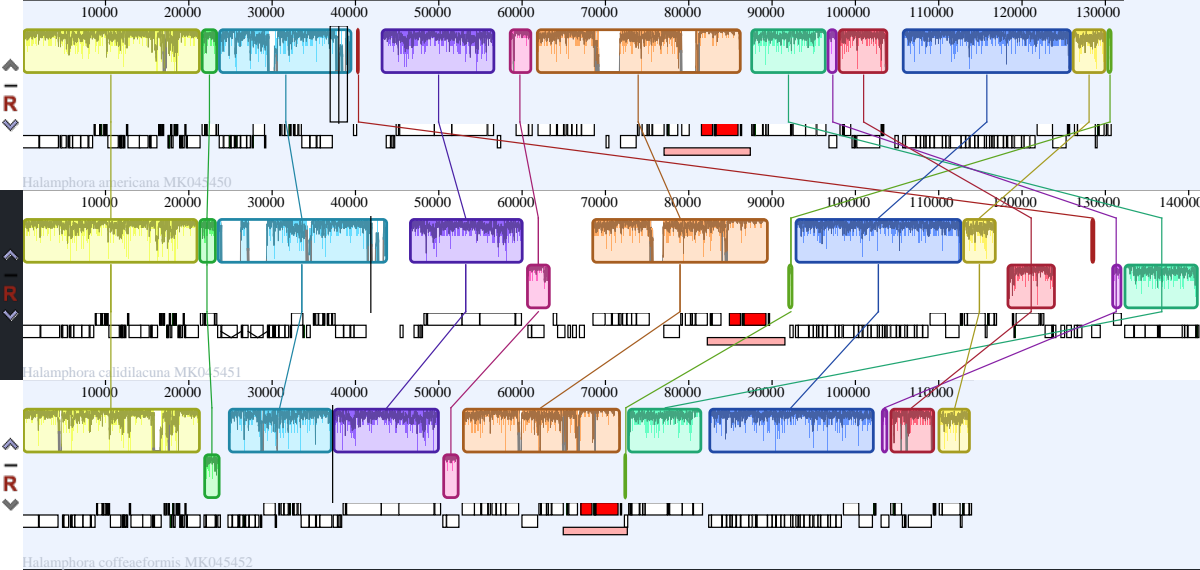

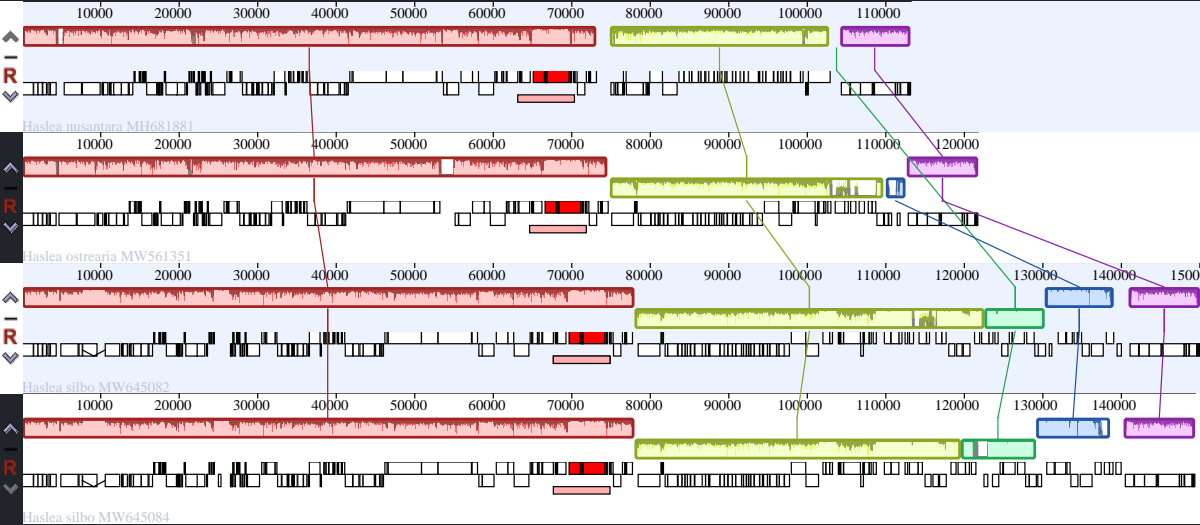

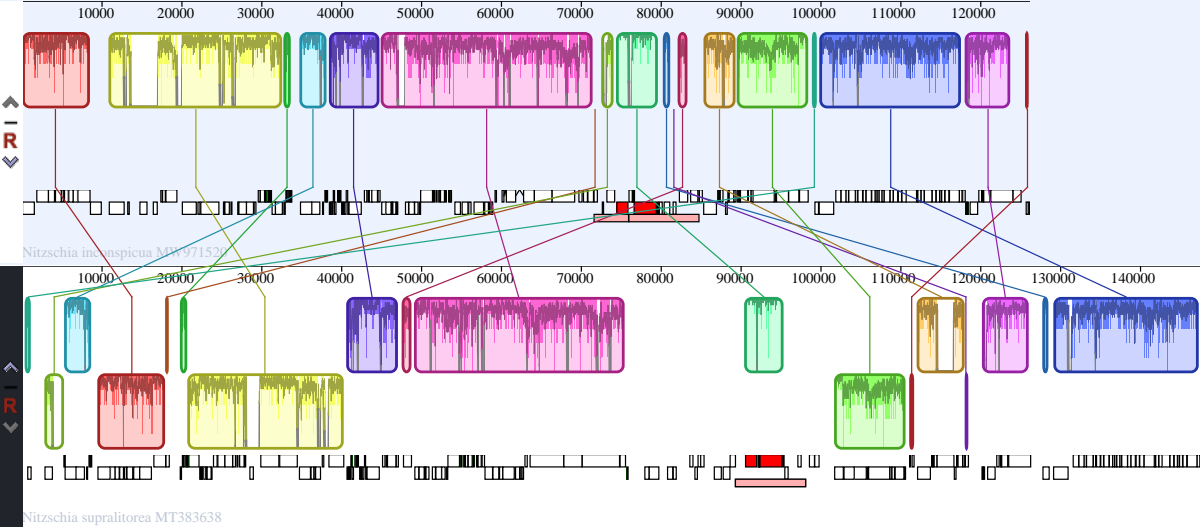

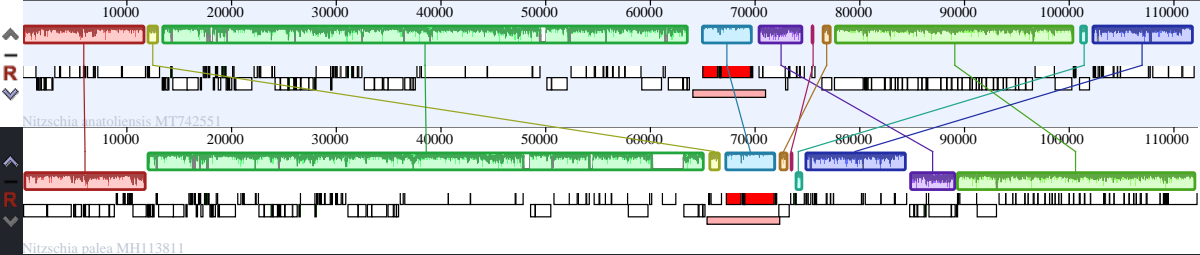

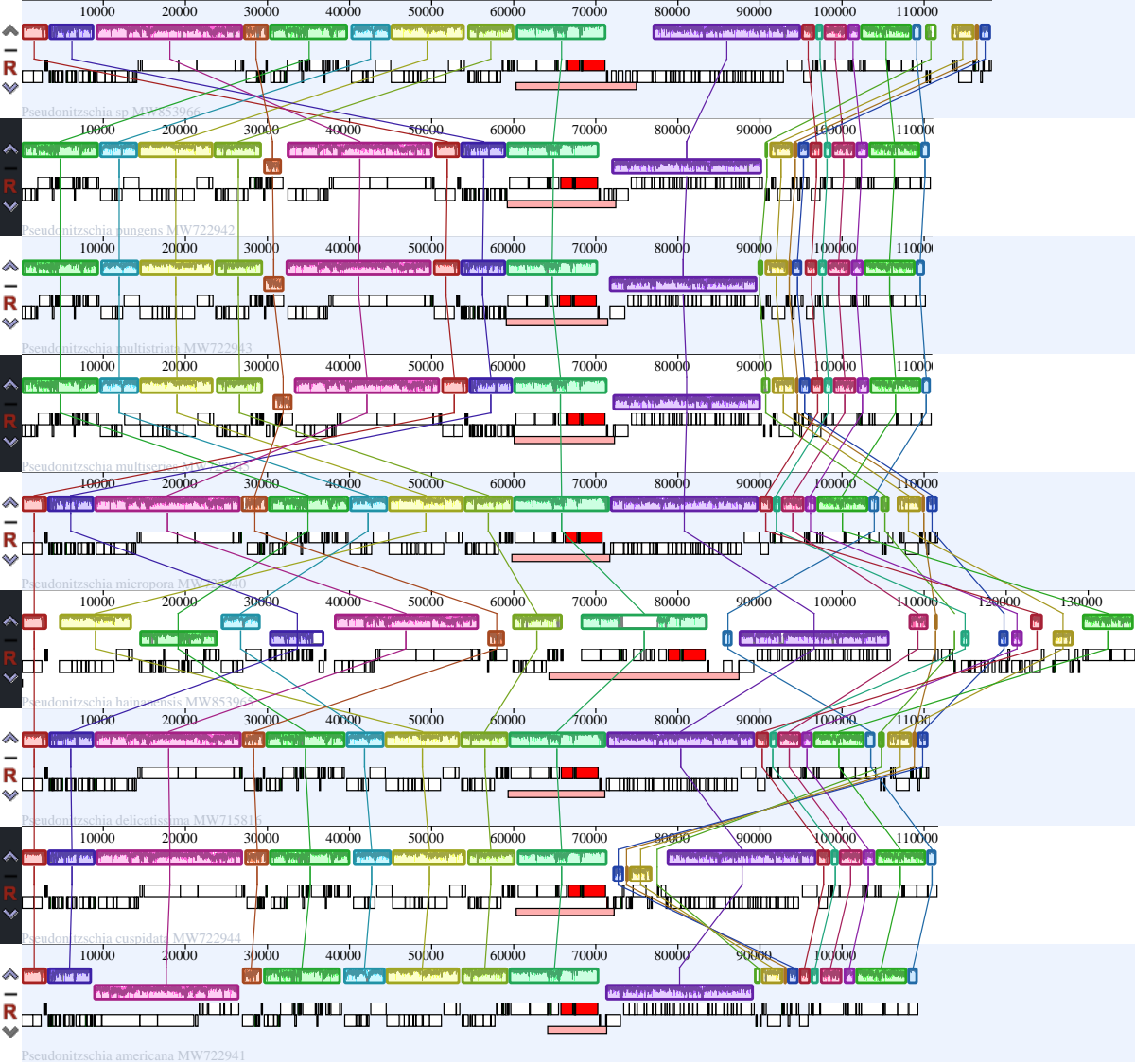

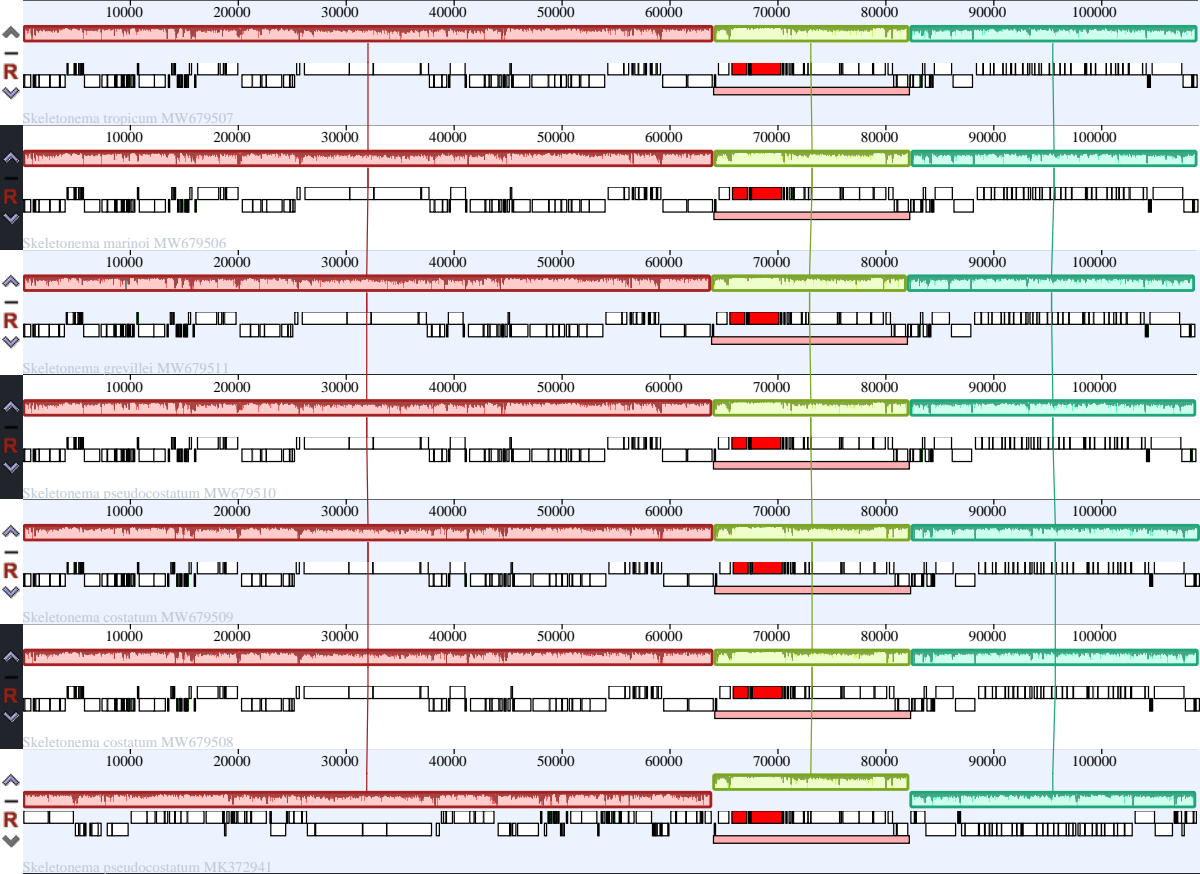

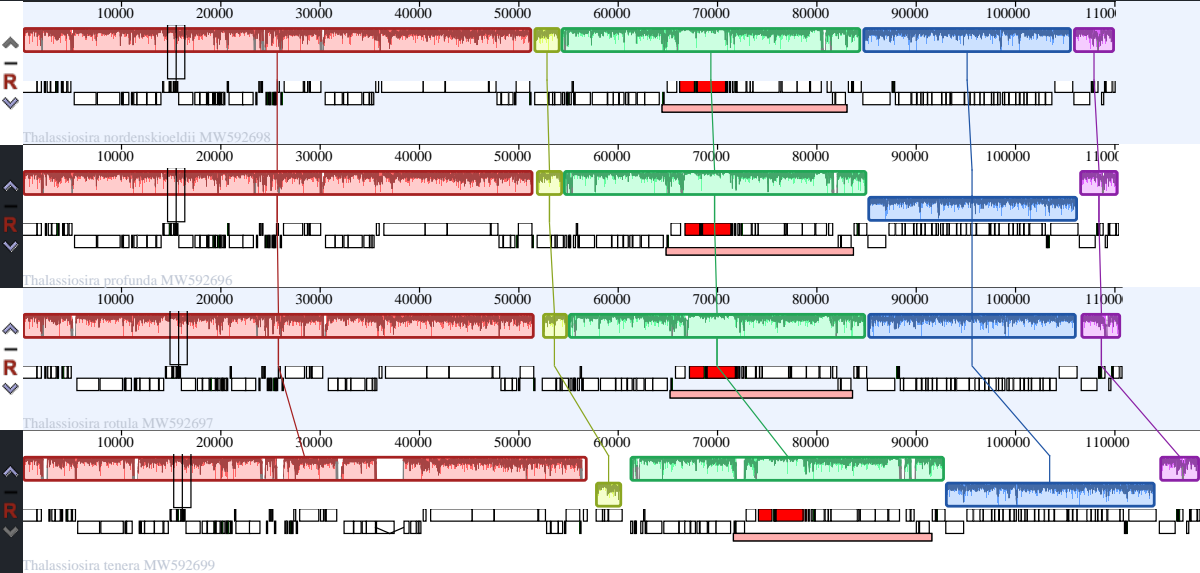

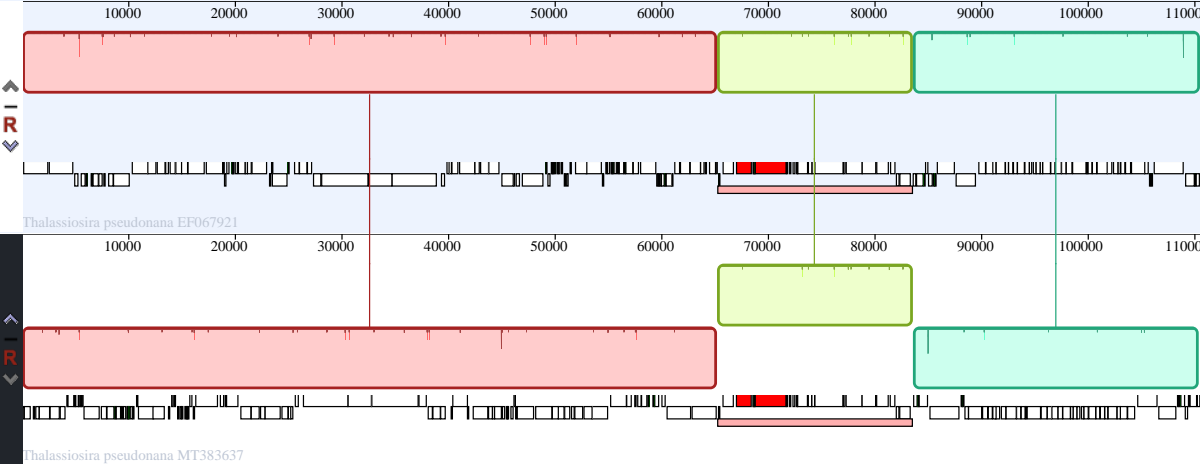

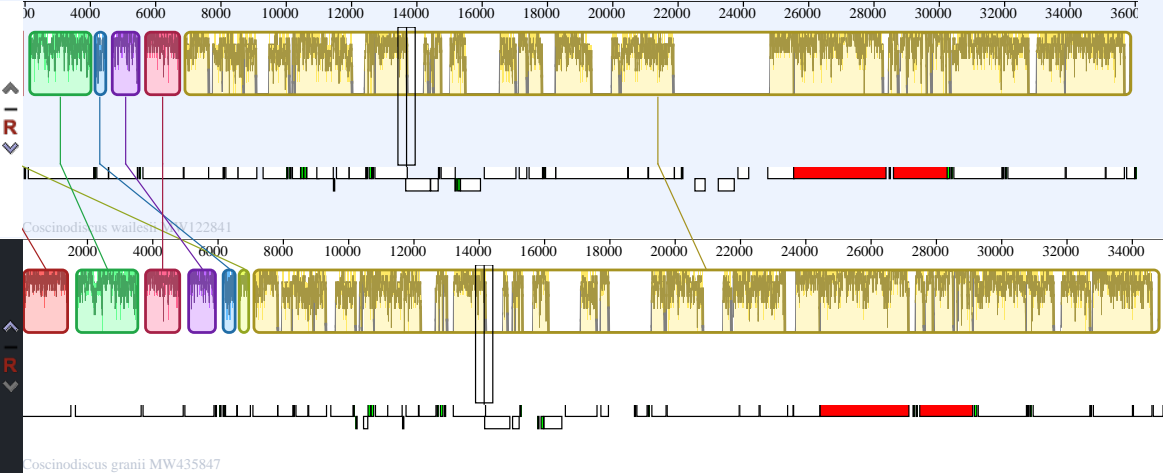

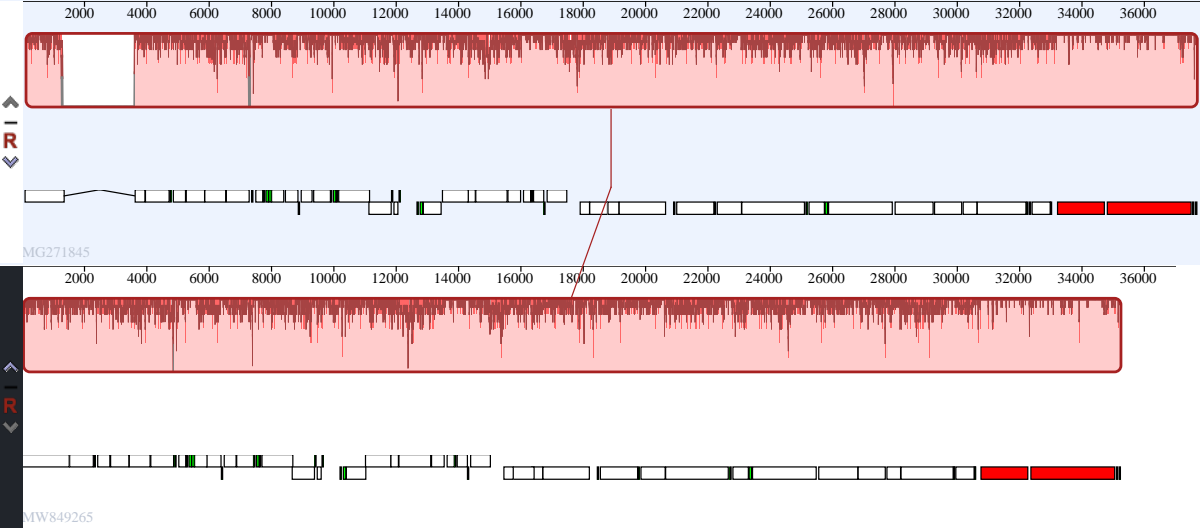

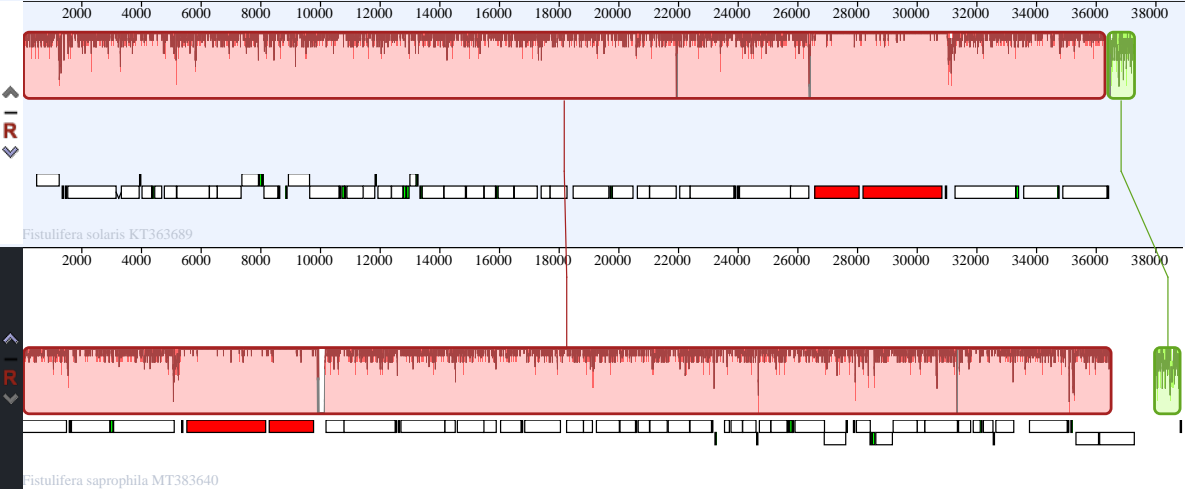

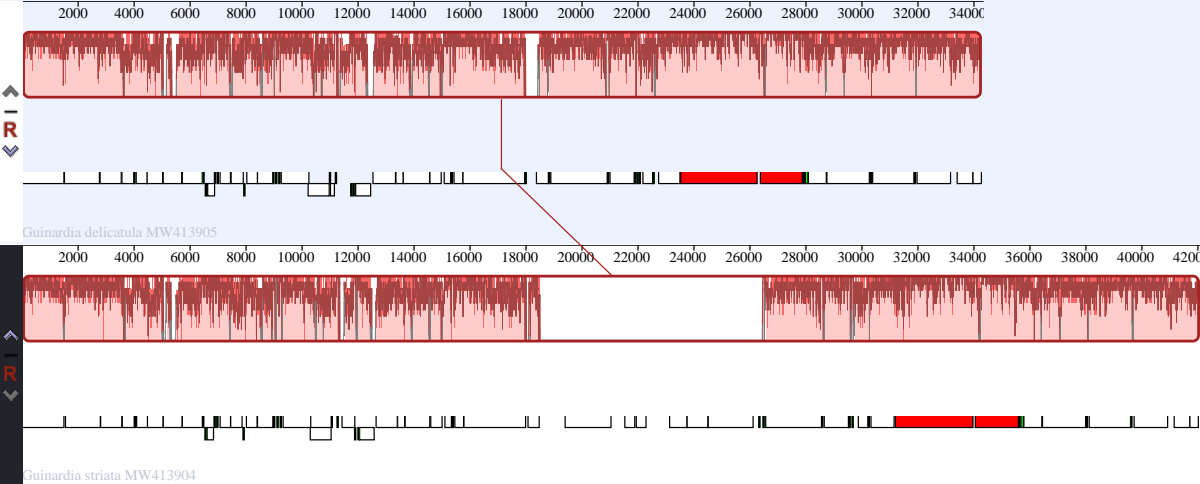

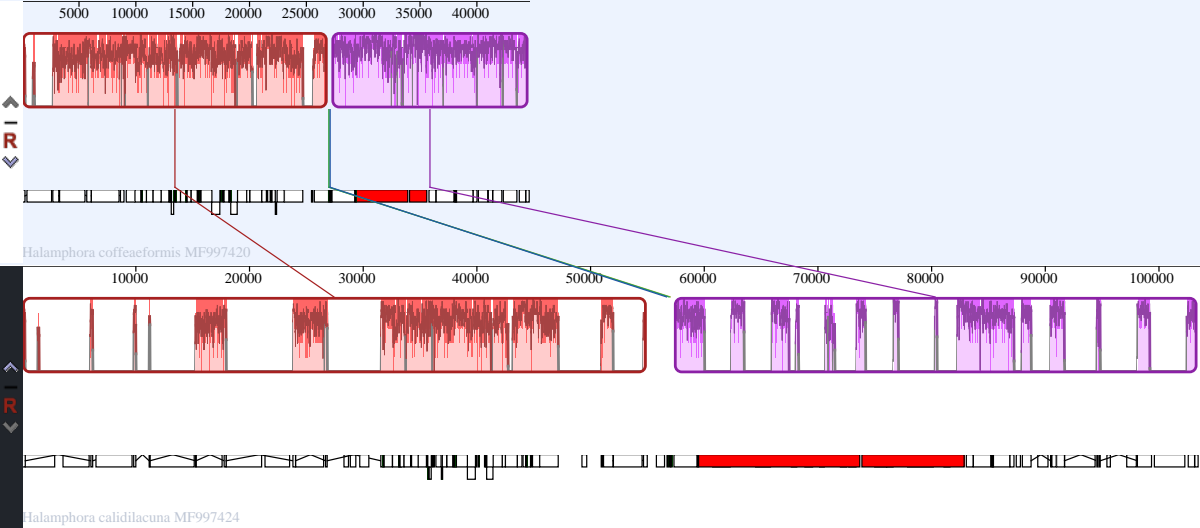

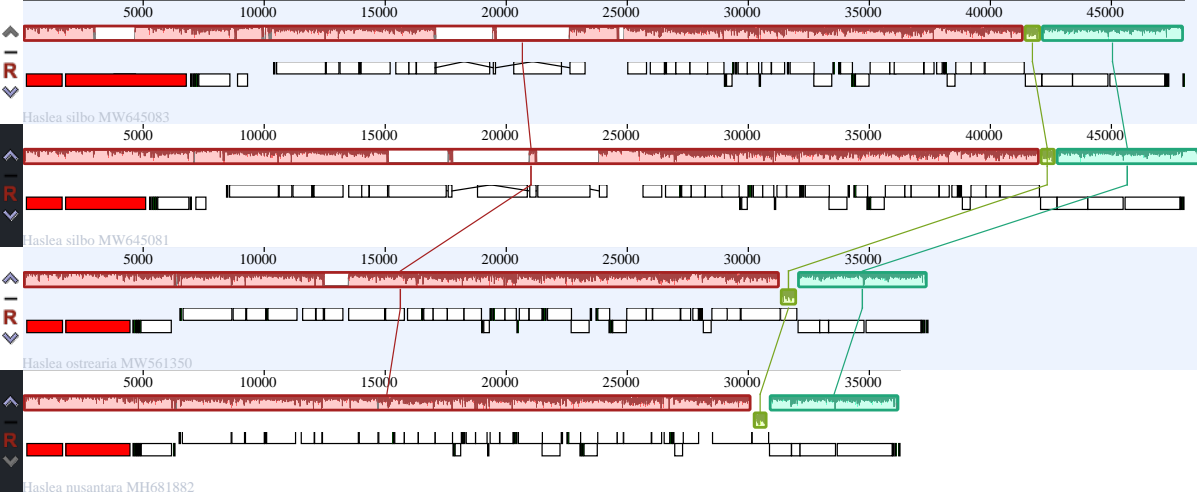

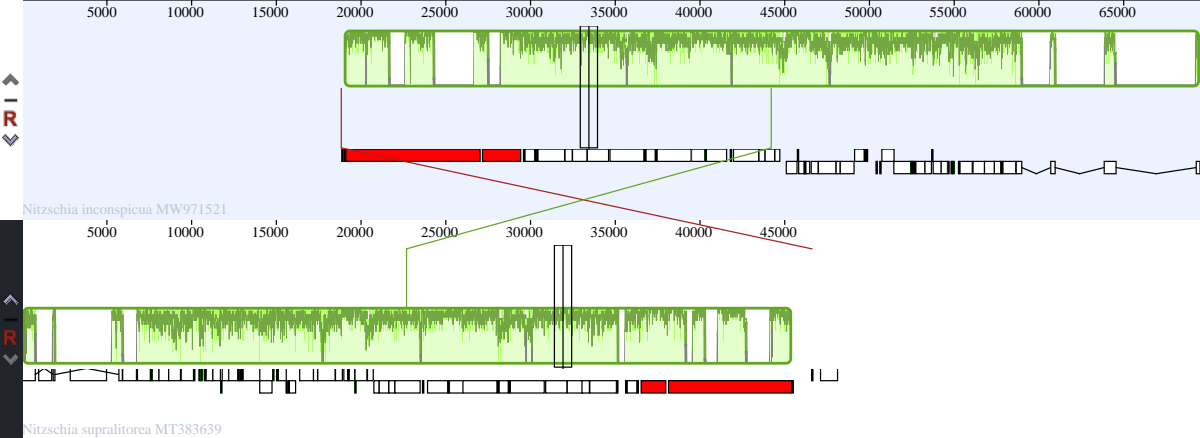

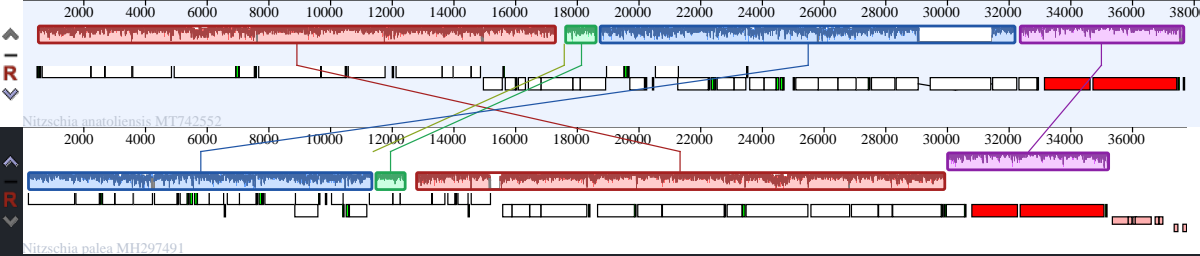

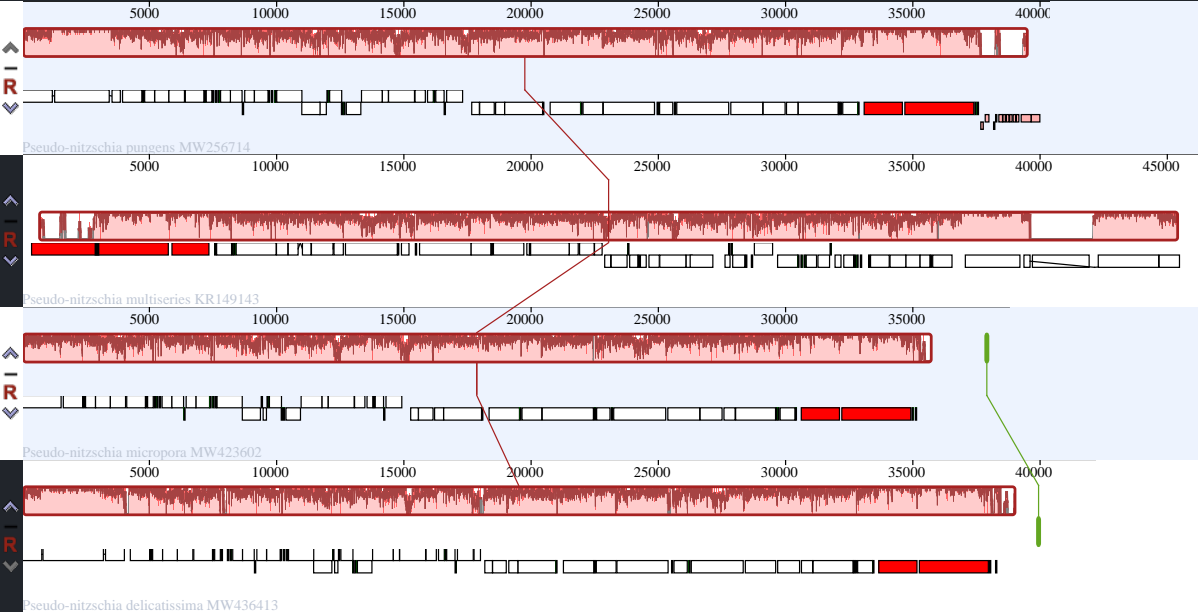

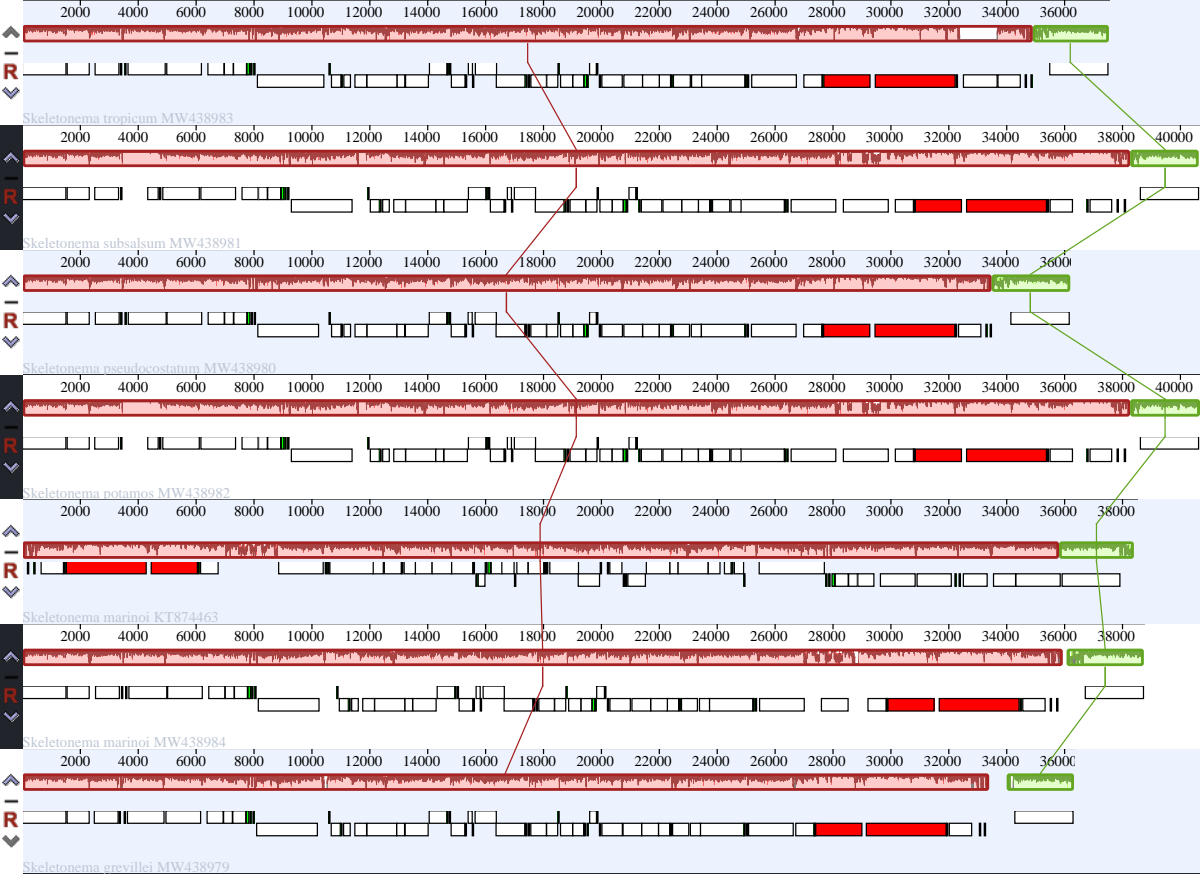

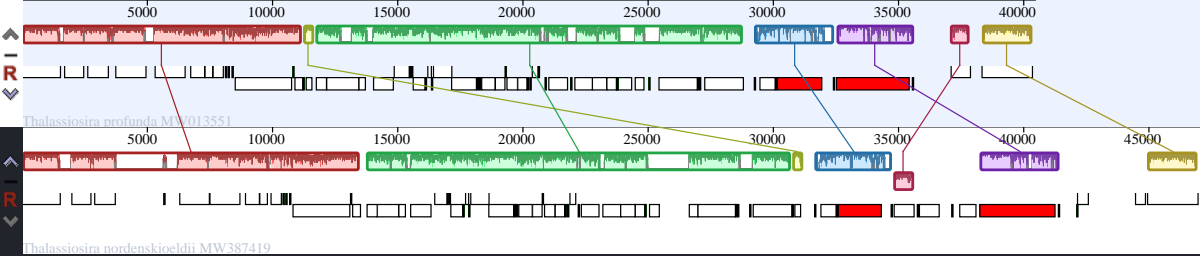

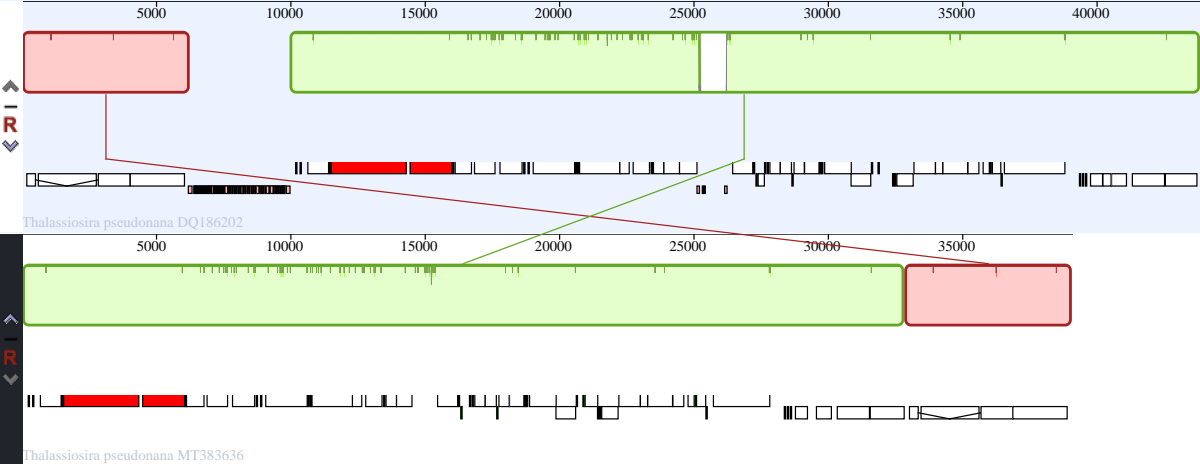

Supplement: S3 File — Compilation of synteny comparisons (higher resolution images) for each genus. (PDF) [file pone.0331749.s006.pdf]
